# Novel Insights into Enhanced Stability of Li‐Rich Layered and High‐Voltage Olivine Phosphate Cathodes for Advanced Batteries through Surface Modification and Electron Structure Design

**DOI:** 10.1002/advs.202413054

**Published:** 2024-12-27

**Authors:** Zhili Liang, Abdulaziz Baubaid, Mariusz Radtke, Maximilian Mellin, Clément Maheu, Sandipan Maiti, Hadar Sclar, Igor Píš, Silvia Nappini, Elena Magnano, Federica Bondino, Robert Winkler, René Hausbrand, Christian Hess, Lambert Alff, Boris Markovsky, Doron Aurbach, Wolfram Jaegermann, Gennady Cherkashinin

**Affiliations:** ^1^ Institute of Materials Science Technische Universität Darmstadt Peter‐Grünberg‐Str. 2 D‐64287 Darmstadt Germany; ^2^ Department of Chemistry Eduard‐Zintl‐Institut für Anorganische und Physikalische Chemie Technische Universität Darmstadt Peter‐Grünberg‐Str. 8 D‐64287 Darmstadt Germany; ^3^ Department of Chemistry Institute for Nanotechnology and Advanced Materials (BINA) Bar‐llan University 5290002 Ramat‐Gan Israel; ^4^ CNR – Istituto Officina dei Materiali (IOM) Strada Statale 14, km. 163,5 in Area Science Park Basovizza 34149 Trieste Italy; ^5^ Nanotechnology Research Laboratory Faculty of Engineering University of Sydney 2006 Camperdown Australia; ^6^ Department of Chemistry BINA – BIU Institute of Nanothechnology and Advanced Materials INIES – Israel National Institute of Energy Storage (supported by Israel Ministry of Energy and Infrastructures) Bar‐Ilan University 5290002 Ramat‐Gan Israel; ^7^ Present address: Nantes Université, CNRS Institut des Matériaux de Nantes Jean Rouxel, IMN F‐44000 Nantes France

**Keywords:** cathode/electrolyte interface, cationic and anionic redox, electrochemical potential and electronic structure, in‐situ and operando, LCP, Lithium‐rich NCM, XANES and RPES and XPS and Raman

## Abstract

The design of cathode/electrolyte interfaces in high‐energy density Li‐ion batteries is critical to protect the surface against undesirable oxygen release from the cathodes when batteries are charged to high voltage. However, the involvement of the engineered interface in the cationic and anionic redox reactions associated with (de‐)lithiation is often ignored, mostly due to the difficulty to separate these processes from chemical/catalytic reactions at the cathode/electrolyte interface. Here, a new electron energy band diagrams concept is developed that includes the examination of the electrochemical‐ and ionization‐ potentials evolution upon batteries cycling. The approach enables to forecast the intrinsic stability of the cathodes and discriminate the reaction pathways associated with interfacial electronic charge‐transfer mechanisms. Specifically, light is shed on the evolution of cationic and anionic redox in high‐energy density lithium‐rich 0.33Li_2_MnO_3_·0.67LiNi_0.4_Co_0.2_Mn_0.4_O_2_ (HE‐NCM) cathodes, particularly those that undergo surface modification through SO_2_ and NH_3_ double‐gas treatment to suppress the structural degradation. The chemical composition and energy distribution of the occupied and unoccupied electronic states at the different charging/discharging states are quantitatively estimated by using advanced spectroscopy techniques, including operando Raman spectroscopy. The concept is successfully demonstrated in designing artificial interfaces for high‐voltage olivine structure cathodes enabling stable battery operation up to 5.1 V versus Li^+^/Li.

## Graphical abstract

The electron energy band diagram concept is developed to predict the intrinsic stability of cathode materials for Li‐ion batteries and to discriminate delithiation/lithiation reactions from interfacial charge‐transfer mechanisms. Specifically, an enhanced structural stability of 0.33Li_2_MnO_3_·0.67LiNi_0.4_Co_0.2_Mn_0.4_O_2_ (HE‐NCM) materials treated with SO_2_ and NH_3_ is explained. An efficient coating for high voltage olivine‐structure cathodes is designed, enabling stable electrochemical cycling.

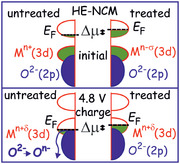

## 1 Introduction

The limited energy density provided by cationic redox in the classical cathode materials for Li‐ion batteries has targeted extensive research on triggering reversible anionic redox to achieve extra capacity.^[^ ^1^, ^2^ ^]^ In conventional lithium transition metal oxides (LMO) of layered structure, the O 2p states hybridized with antibonding M 3d states are situated further from the Fermi level (*E*_F_) than M 3d states that requests a higher voltage (>4.2 V vs Li^+^/Li) to trigger the anion redox. An electron release from the O 2p states of the lattice is generally an irreversible process for the inverse O^–^ → O^2−^ chemical reaction. In parallel to that, the partially oxidized O^2−^ ions of the lattice oxygen can leave the solid in gaseous form, thereby determining the intrinsic voltage limit of the first‐generation cathode materials.^[^ ^3^ ^]^ The situation is different for Li‐rich cathodes (LRC) where the structurally modified electronic configuration facilitates anion redox chemistry, which is believed to enable reversible oxygen redox.^[^ ^4^, ^5^, ^6^ ^]^ A most known approach for reversible anionic redox is the chemical synthesis of LRC via the integration of Li_2_MnO_3_ and LiMO_2_ (M = transition metal) phases,^[^ ^7^ ^]^ where an excess of lithium composition is thermodynamically favorable to substitute the transition metal in the MO_2_ layers by Li‐ions.^[^ ^5^, ^6^ ^]^ The oxygen redox is activated by delithiation of LRC at ≥ 4.5 V when the Fermi level crosses the top electronic states of Li_2_MnO_3_ which are mostly contributed from oxygen, whereas Mn 3d is situated at higher energy.^[^ ^8^ ^]^ The accompanied electron removal from the O 2p level leads to the formation of Li_2_O with its partial release from Li_2_MnO_3_ without the structural destabilization of the lattice inherent for conventional layered oxides.^[^ ^9^ ^]^ According to density functional theory(DFT) calculations, a lithium excess in LRC leads to the formation of a non‐bonding, non‐hybridized O 2p orbital, which is structurally positioned within the Li−O−Li bond and electronically between the antibonding M 3d state and the O 2p bonding state of the lattice oxide.^[^ ^6^ ^]^ Energetically, the non‐hybridized O 2p orbital is more prone to donate an electron due to a closer energy to the *E*_F_ in comparison with the O 2p orbital bonded to M 3d states. Moreover, triggering the anionic redox associated with the non‐hybridized O 2p orbital does not lead to the structural destabilization of the lattice due to the localized character of this oxygen orbital.

Nevertheless, along with anomalous high capacity, LRCs are characterized by various structural changes such as layered‐to‐spinel transformation upon cycling followed by the formation of an electrochemically inactive phase with a rock‐salt type disordered structure responsible for degradation, notable voltage hysteresis and long‐term voltage fade.^[^ ^10^ ^]^ Since the given phase transitions begin typically from the surface accompanied by oxygen release,^[^ ^11^, ^12^ ^]^ several approaches have been developed to design the surface conditions suppressing structural degradation.^[^ ^13^ ^]^ These include chemical treatment of LRC with various solutions and gases,^[^ ^14^, ^15^, ^16^ ^]^ doping of the surface layers,^[^ ^17^ ^]^ incorporation of an anion counterpart into oxygen site,^[^ ^18^ ^]^ etc. Conceptually, the role of the modified surfaces is exclusively considered as a protective layer against the involvement of the cathodes into chemical reactions with the electrolytes and the oxygen release, or as Li‐ion conducting layer facilitating Li^+^ transport across the electrode‐electrolyte interfaces. However, the redox activity of the modified surfaces is often neglected thereby greatly underestimating the importance of the bulk‐surface‐interface to electrolyte electronic charge transfer mechanisms affecting both an extra capacity and the internal stability of LRC.

Among the high‐energy density cathode materials, Li‐rich transition metal oxides with the chemical formula of *x*Li_2_MnO_3_·(1−*x*)Li(M)O_2_ (M = Mn, Ni, Co; *x* < 0.5) has drawn attention due to their high discharge capacity (>200 mAh g^−1^) and environmental friendliness. 0.33Li_2_MnO_3_•0.67LiNi_0.4_Co_0.2_Mn_0.4_O_2_ (named as HE−NCM) provides a discharge capacity >250 mAh g^−1^. A higher capacity retention and rate capability, low voltage hysteresis and low impedance can be further achieved via the thermal surface treatment of HE−NCM with SO_2_ and NH_3_ gases studied in our previous work.^[^ ^19^ ^]^ Such solid‐gas reactions modify the surface of the cathode to a depth of several nanometers,^[^ ^20^ ^]^ mostly impacting the surface electronic configuration and chemical composition,^[^ ^21^ ^]^ that might lead to more complex charge transfer mechanisms upon charging the HE‐NCM in comparison with the untreated counterpart or with the bulk related redox processes.

In this contribution, we propose a novel concept based on the electron energy band diagrams approach to shed light on the cationic and anionic redox within the modified surface. We study the evolution of the electronic structure and interfacial chemical composition coupled to the examination of the electrochemical potential shift across the M 3d‐ and O 2p‐ occupied states caused by delithiation/lithiation of a series of HE−NCMs upon their cycling to ≥ 4.8 V (versus Li^+^/Li). For this purpose, we apply *quasi*‐*in‐situ* surface sensitive electron spectroscopy techniques, combined with theoretical calculations of X‐ray absorption spectra and operando Raman spectroscopic experiments to probe the structure at the surface and in the bulk of the cathodes. Such a complementary techniques approach enables to explain the relevant electrochemical reactions, charge transfer mechanisms, and the nature of distortions at the oxygen site occurred at the modified surfaces and interfaces upon electrochemical cycling of various high‐voltage cathodes for Li ion batteries.

## 2 Results and Discussion

HE‐NCMs studied here are: a) *untreated HE‐NCM*, b) *treated HE‐NCM*, c) *untreated HE‐NCM composite* = *untreated* cathode, d) *treated HE‐NCM composite* = *treated* cathode (see the Experimental section for the details).

### 2.1 Oxidation States of the Cations and Anion at the Surface of As‐Prepared Cathode Materials

**Figure 1:**
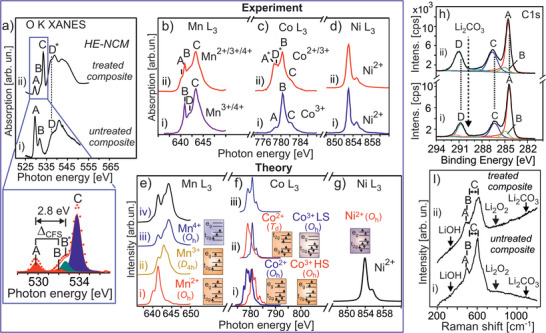
a) O K‐edge XANES of HE‐NCM prepared under different conditions: *untreated composite* (i) and *treated composite* (ii) obtained in the total electron yield (TEY) mode. The insert shows the zoomed area of O K XANES in the 528 – 537 eV range after the background subtraction and fitting the spectrum; Δ_CFS_ is crystal field splitting. b–d) Mn L_3_ (b), Co L_3_ (c), and Ni L_3_ (d) XANES of the *untreated composite* (i), *treated composite* (ii). e) The calculated Mn L_3_ XANES of Mn^2+^ state in *O*_h_ symmetry (i), Mn^3+^ state in *D*_4h_ symmetry (ii), Mn^4+^ state in *O*_h_ symmetry (iii); linear combination of Mn^3+^ (13%), Mn^2+^ (20%) and Mn^4+^ (67%) (iv). f) Calculated Co L_3_ XANES with Co^2+^ (high‐spin, HS) state (blue) and Co^3+^ (HS) (red) in *O*_h_ sites (i); Co^2+^ (HS) in the tetrahedral (*T*_d_) (red) and Co^3+^ (low‐spin, LS) in *O*_h_ (blue) symmetries (ii); linear combination of Co^2+^ HS in *T*_d_ (44%) and Co^3+^ LS in *O*_h_ (56%) (iii). g) The calculated Ni L_3_ XANES for Ni^2+^ in *O*_h_ symmetry. h, I) C 1s photoelectron spectra (hν = 1486.7 eV) (h) and Raman spectra (hv = 632 nm) (I) of as‐prepared: *untreated HE‐NCM composite* (i) and *treated HE‐NCM composite* (ii). h) The most intense features are ascribed to C‐C (A = 284.5 ± 0.1 eV), C‐H (B = 285.0 ± 0.1 eV), C‐O (C = 286.4 ± 0.2 eV), and C‐F (from PVDF, D = 290.9 ± 0.2 eV). I) The vertical arrows show the expected band positions of LiOH, Li_2_O_2_ and Li_2_CO_3_. Note however, that these species are not detected in our Raman spectroscopic experiments. The vertical lines indicate vibrations associated with Li_2_MnO_3_ (A ≈ 488 cm^−1^) and LiMO_2_ (B = 491 – 497 cm^−1^ and C = 518 – 614 cm^−1^).

Soft X‐ray photoelectron spectroscopy (SPES) and X‐ray absorption spectroscopy (XAS) probe depth of a few nanometres giving a unique opportunity to study the surface evolution and its involvement in the cationic‐ and anionic‐ redox processes. The O K‐edge X‐ray absorption near‐edge structure (XANES) occurs due to an electron transition from the O 1s core‐level to unoccupied O 2p states hybridized with empty M 3d electronic states in the 529−535 eV photon energy range.^[^ ^22^ ^]^ The wide band above 535 eV is associated with the O 1s to M 4s‐ and 4p‐ unoccupied states electron transition (**Figure** **1**a). In the *untreated HE‐NCM*, the oxidation and spin states of transition metals are Mn^4+^ (high‐spin, HS), Co^3+^ (low‐spin, LS) and Ni^2+^ (see the M 3d electronic configuration in details in Figure S1a‐i,v,ix, Supporting Information).^[^ ^21^ ^]^ Accordingly, the t_2__g_ (lowest energy) and e_g_ (highest energy) electronic levels of M 3d (labeled as A and B in the O K‐edge, Figure 1a‐i) are bound to the O 2p states in an octahedral (*O*_h_) symmetry.^[^ ^21^ ^]^ For the ground state electronic configuration, the t_2__g_ orbitals of the Co^3+^ and Ni^2+^ states are fully occupied. Therefore, the A peak (529.6 eV) can be associated only with the Mn^4+^ (t_2__g_) empty state (Figure S1a‐i, Supporting Information). The empty e_g_ states of Mn^4+^, Co^3+^, and Ni^2+^ (partially) (Figure S1a‐i,v,ix, Supporting Information) mostly contribute to the B peak at 531.8 eV. The energy difference between the A (t_2__g_) and B (e_g_) peaks defined by crystal field splitting (Δ_CFS_), gives Δ_CFS_ = 2.2 eV in a good agreement with Δ_CFS_ ≈ 2.4 eV inherent for tetravalent metal ions bound to oxygen p state in *O*_h_ symmetry.^[^ ^23^ ^]^ The relevant ground state electronic configuration (with almost negligible contribution from Mn^3+^ (Figure 1b‐i) is also evidenced by M L_3,2_‐ edges XANES, which occurs as dipole allowed electron transitions from the M 2p core level to the M 3d empty states (Figure 1b‐i–d‐i). The conclusion on oxidation states of M is in agreement with theoretical calculations of the Mn L_3_, Co L_3_ (Co^3+^, LS) and Ni L_3,2_ XANES (Figure 1e‐iii,f‐ii,g) and the experimental data obtained on a model NCM thin‐film cathode (Figure S1b‐ii–d‐ii, Supporting Information).

The SO_2_ and NH_3_ double gas treatment modifies the electronic configuration at the surface of HE‐NCM. The purpose of such a chemical treatment was discussed in our previous works.^[^ ^15^, ^21^ ^]^ A brief discussion on the surface modification with either SO_2_ and NH_3_ alone can be found in the Discussion Section S1 (Supporting Information). The double gas treatment of HE‐NCM induces numerous modifications observed in the O K XANES (Figure 1a‐ii), a decrease in the A and B peaks intensity while the peak C emerges and two minor features (D^*^ and B^*^) also appear. The A and B decrease evidences an increasing the M 3d occupation due to a reduction of transition metal ions that would have a trend in lowering the M 3d – O 2p hybridization and a smaller crystal field splitting for *O*_h_ symmetry.^[^ ^23^ ^]^ However, the estimated Δ_CFS_ = 2.8 eV is higher than expected value for both the *O*_h_ and tetrahedral (*T*_d_) symmetries (it should be noted that Δ_CFS_ for *T*_d_ is typically lower than that of an *O*_h_ environment). A more reasonable assumption is an additional empty state contributing to the peak B. This “hidden” state is resolved via spectral decomposition of the O K XANES followed by a weighted least‐squares fitting of the spectral region (Figure 1a‐ii, insert). Accordingly, the empty state B (532.0 eV) is assigned to the M 3d (e_g_) state giving Δ_CFS_ = 2.3 eV. The B^*^ feature at higher energy (532.7 eV) is most probably associated with vacancies in the nitric oxide (NO) species formed in a small amount due to chemical reactions of NH_3_ with the HE‐NCM surface.^[^ ^15^ ^]^ The empty O 2p state of N−O is typically manifested at 532.7 eV in O K XANES.^[^ ^23^ ^]^ In spite of a weak adsorption of NH_3_ onto the HE‐NCM surface,^[^ ^21^ ^]^ our previous research showed that ammonia modifies the electronic structure of the cathodes significantly, acting as a reducing agent,^[^ ^15^ ^]^ which in turns leads to the formation of NO_x_ (Figure S2‐i, Supporting Information). The shoulder D^*^ in the O K XANES in the 537.5 – 538.5 eV range (Figure 1a‐ii is ascribed to SO_4_^2−^ groups,^[^ ^24^, ^25^ ^]^ in accordance with the O 1s (≈ 532 eV) and S 2p (169.4 eV) photoelectron emissions of the *treated HE‐ NCM composite* (Figures S3‐iii and S4, Supporting Information).^[^ ^21^ ^]^ A reduction of the oxidation states of transition metals caused by SO_2_ and NH_3_ double gas treatment is also detected by the Mn L‐ and Co L‐ XANES with the clear visible superposition of Mn^4+^ with Mn^3+^/Mn^2+^ and Co^3+^/Co^2+^ oxidation states, whereas Ni remains mostly in 2+ state (Figure 1b‐ii,c‐ii,d‐ii). The coordination of transition metals with oxygen at the surface of the *treated HE‐NCM* was explored by theoretical calculations of the Mn L_3_‐ and Co L_3_‐ edges XANES in various environments of the M 3d states with the O 2p level. These calculations show that the experimental Mn L_3_ and Co L_3_ XANES are well described by a linear combination of the *O*_h_‐ and *D*_4h_‐ symmetries and of the *O*_h_‐ and *T*_d_‐ symmetries, respectively (Figure 1e‐iv,f‐iii). Note, that *T*_d_ symmetry is often assigned to spinel structure, whereas *D*_4__h_ symmetry is a reduction of *O*_h_ symmetry due to Jahn‐Teller effect. More interesting is the peak С (533.7 eV) which emerges in O K XANES *only* after the double gas treatment of HE‐NCM (Figure 1a‐ii). Lithium carbonate, which has a similar energy, can be ruled out, because the chemical composition of the carbon species is very similar for both the *untreated*‐ and *treated*‐ *HE‐NCM composites* (Figure 1h). The absence of Li_2_CO_3_ in the bulk is also confirmed by Raman spectroscopy (Figure 1I–ii). Thus, O K XANES at 533.7 eV is assigned to the surface Li_2_O. A more discussion on other oxide species appears in the Discussion Section S2, Supporting Information). In the as‐prepared *untreated HE‐ NCM* *composite*, Li_2_O associated with a Li−O−Li bond is not detected neither by the surface sensitive techniques nor by operando Raman spectroscopy which probes the subsurface/bulk of the samples (Figure 11a–i,I–i; Figure S5, Supporting Information).

### 2.2 Distribution of Electronic States Near the Fermi Level as a Predictor of Redox Mechanisms

**Figure 2:**
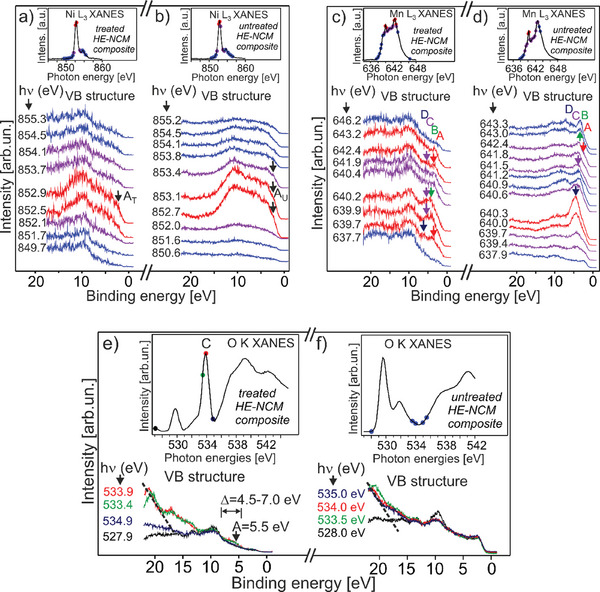
Valence band (VB) photoemission spectra of *HE‐NCM treated‐* (a,c,e) and *untreated‐* (b,d,f) *composites* collected at photon energies of the 849 eV – 856 eV (a, b), 637 eV – 646 eV (c, d), 527.9 eV (off‐resonance of O K‐edge XANES) (e, f) and the 533.4 – 534.9 eV (on‐resonance for the C peak in O K‐edge XANES of the *treated cathode*). The solid circles in the inserts show excitation energies. The VB spectra collected at the Ni L_3_ ‐edge (a, b) of the highest intensity (in red) show the Ni^2+^ 3d resonance at a) A_T_ = 2.5 eV and b) A_U_ = 2.35 eV for the *treated*‐ and *untreated*‐ *HE‐NCM*, respectively. The VB spectra collected at the Mn L_3_ ‐edge of the *treated HE‐NCM* (c) show the two resonance structures (highlighted in red) at ≈ 640 and ≈ 642.7 eV photon energies, where A = 3.6 ± 0.1 eV, B = 4.1 ± 0.05 eV, C = 5.3 ± 0.1 eV and D = 6.2 ± 0.2 eV are associated with the Mn 3d levels of Mn^2+^, Mn^3+^ and Mn^4+^ oxidation states. d) The resonances at A = 2.5 ± 0.2 eV, B = 3.4 ± 0.4 eV, C = 3.7 ± 0.2 eV and D = 4.4 ± 0.2 eV in the *untreated HE‐NCM* are mostly with Mn^4+^ state. e) The resonance in the Δ  = 4.5 – 7.0 eV binding energy range (labelled by vertical lines and horizontal double arrow) of the *treated HE‐NCM* is mostly assigned to a Li_2_O state. (e, f) The O (KVV) Auger transition contributed at *E*_bin_ >  5 eV is shown by the dashed line.

Quantitative energy band diagrams are crucial to understand the underlying mechanisms occurring during the double SO_2_ and NH_3_ gas treatment. Moreover, understanding of the distribution of electronic states near *E*_F_, coupled to an ability to monitor changes in the electrochemical potential of the cathodes (µ_C_^e^ = *E*_F_) upon electrochemical cycling of batteries, is important to predict the involvement of the cations and the anion into redox reactions. In our work, the energy distribution of the occupied electronic states near *E*_F_ is derived from the results of resonant photoemission spectroscopy (RPES). By collecting the valence band photoelectron spectra while scanning the photon energies across the M L‐ and O K – edges, it is possible to observe the resonances of the M 3d‐ and O 2p‐ valence states. These resonances arise from a quantum mechanical interference between the direct photoemission process and the recombination of an excited state.^[^ ^26^ ^]^ Our RPES experiments evidence three states closest to *E*_F_. One is associated with Co^2+^ in the *treated* cathode (A^1^_T_ = 1.7 eV ± 0.1 eV), whereas Co^3+^ at A^2^_T_ = 2.0 ± 0.1 eV and A_U_ = 1.9 ± 0.1 eV is revealed for the *treated*‐ and *untreated*‐ cathodes, respectively (Figure S6, Supporting Information). The Ni^2+^ 3d level is situated at higher energies, *E*_bin_ = 2.3 – 2.5 eV for both cathodes (**Figure** **2**a,b). The presence of the Mn^4+^/Mn^3+^/Mn^2+^ oxidation states in the *treated* cathode leads to the several resonance features of Mn 3d states (Figure 2c,d). These states are more distant from *E*_F_ as compared to the Ni 3d and Co 3d (Figure 2a,b; Figure S6, Supporting Information) and have a higher binding energy than Mn^4+^ of the *untreated* cathode (Figure 2c,d). The more pronounced resonances of the M 3d occupied states of the *untreated* HE‐NCM might indicate that the electrons of the transition metals are more localized in this cathode as compared to the *treated* counterpart (the mapping of photon energies versus binding energies is shown in Figure S7, Supporting Information).

The O 2p resonance shows the general characteristics for both cathodes in the range of 9 – 11 eV (Figure 2e,f). A slight extension of the O 2p state to higher binding energies in the case of *treated HE‐NCM composite* is associated with the O 2p – M 3d bonding hybrid states. The main difference in the valence band structure associated with the O 2p states of the two cathodes is the resonance for the *treated* cathode (absent in the *untreated* cathode, Figure 2f) with an onset at *E*_bin_ ≈ 4.5 eV and the maximum at *E*_bin_ = 5.5 eV at photon energy of ≈ 534 eV (Figure 2e; Figure S8a,c, Supporting Information). This occupied O 2p state is mostly assigned to Li_2_O (see above our discussion on the nature of the *C* peak in the O K XANES) in agreement with the previously reported *E*_bin_ values for lithium oxide lying in the ≈ 4.5 – 10 eV range.^[^ ^27^ ^]^ The lack of the relevant O 2p resonance in the *untreated HE‐NCM composite* (Figure 2f; Figure S8b, Supporting Information) makes the presence of Li_2_O unlikely in the absence of the SO_2_ and NH_3_ surface treatment. LiOH is expected at a slightly higher binding energies (7–10 eV,^[^ ^28^ ^]^) and is not detected neither for the *treated‐* or *untreated*‐ cathodes (Figure 2e,f).

Thus, our RPES results show that the binding energy sequence of the M 3d (t_2__g_) occupied states hybridized with the O 2p (lattice) are in the following order: Mn > Ni > Co. Also, the O 2p occupied state of Li_2_O of the *treated HE‐NCM* is situated at lower binding energy from the O 2p state of the lattice oxygen. The energy distribution of the unoccupied electronic states near *E*_F_, obtained from the O K‐, Co L‐, Ni L‐, and Mn L‐ edges XANES, is plotted on a unified energy scale (Figure S9a,b, Supporting Information). It is worth noting that the L‐edges do not directly represent the density of states (DOS) due to a complex impact of the final‐state effects on the shape of L‐edge.^[^ ^29^ ^]^ Nevertheless, the representation of M L XANES with respect to *E*_F_ provides valuable insights into the relative energy positions of unoccupied M 3d states.^[^ ^30^, ^31^ ^]^

By neglecting the band bending of the electronic states, which can impact the relative energy positions of the DOS,^[^ ^31^ ^]^ one can conclude that the occupied electronic states of the *treated* cathode are shifted to higher binding energies by ≈ 0.2 eV as compared to the *untreated* cathode, evidenced by the valence band maxima (VBM) at 1.1 ± 0.1 and 0.85 ± 0.1 eV versus *E*_F_ for the *treated‐* and *untreated‐ HE‐NCM composites*, respectively (Figure S10a‐i,b‐i, Supporting Information). The observed VBM shift in the *treated* cathode reflects lifting up of *E*_F_ toward the unoccupied states and is not attributed to the chemical shift. The SO_2_ and NH_3_ double gas treatment causes the reduction of Co^3+^ → Co^2+^ and Mn^4+^ → Mn^2+/3+^. In turn, a partial occupation of the Mn 3d (e_g_)‐ and Co 3d (e_g_)‐ empty states (see the electronic configuration in Figure S1a‐i–vi, Supporting Information) leads to a shift of *E*_F_ away from the VBM, i.e., the photoelectron spectra are shifted to higher binding energies as compared to the *untreated* counterpart. For the *untreated HE‐NCM composites*, the relevant 3d (e_g_) levels are empty (Figure S8a‐i,v, Supporting Information) that explains the *E*_F_ position closer to the VBM.

**Figure 3:**
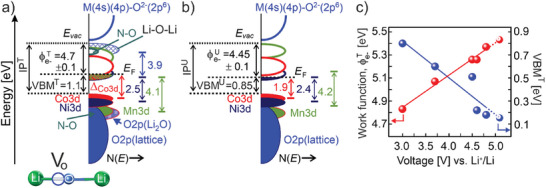
The energy band diagrams of as‐prepared *treated‐* (a) and *untreated‐* (b) *HE‐NCM* cathodes. The electronic states of Ni, Co, and Mn are presented in dark blue, red, and green, respectively. Li−O−Li bond with a vacancy at oxygen (V_o_) is also shown (a). The main difference in the energy diagram of the *treated* cathode is oxygen vacancies in Li_2_O and the presence of N−O bond. Δ_Co3d_ = 1.85 eV ± 0.15 eV for *treated* cathode is an average value of the binding energy of the Co^2+^ 3d (*E*_bin_ = 1.7 eV) and Co^3+^ 3d (*E*_bin_ = 2.0 eV) states with respect to *E*_F_ (see RPES in Figure S6a, Supporting Information for details). VBM^T^, VBM^U^, ϕ_e‐_^T^, and ϕ_e‐_^U^, are the valence band maximum and the work function of the *treated* and *untreated* cathodes, respectively. The VBM and work function were measured at hν = 1486.7 eV and hν = 47 eV, respectively. The estimated values of the ionization potential at these photon energies are IP^T^ = 5.8 ± 0.2 eV and IP^U^ = 5.3 ± 0.2 eV for the *treated‐* and *untreated‐* cathodes, respectively. The increase of work function and ionization potential after the SO_2_ and NH_3_ treatment is assigned to the formation of Li_2_O. The downward band bending (i.e., away from the *E*_F_ in the bulk) of γ ≈ 0.4 eV was observed by probing the electronic structure with higher photon energies (**Table** **1**). For the *treated* cathode, a more colored area near the empty states (as compared to the *untreated* cathode) reflects the *E*_F_ shift toward the unoccupied states due to their partial occupation due to the Mn^2+/3+^ and Co^2+^ states. The work function is different also due to the presence of Li_2_O and N‐O species at the surface. c) Evolution of the work function, ϕ_e‐_^T^, and valence band maximum (VBM^T^) upon charging the *treated* cathode. ϕ_e‐_^T^ and VBM^T^ are labeled by red‐ and blue‐color balls, respectively. The dashed line shows a linear extrapolation of ϕ_e‐_^T^ and VBM^T^ to charging state of 5.1 V.

The electron energy band diagrams of the occupied‐ and unoccupied‐ electronic states for the as‐prepared *treated*‐ and *untreated*‐ *HE‐NCM composites* derived from the current photoemission experiments are shown (**Figure** **3**a,b).

### 2.3 Evolution of the Electrochemical Potential, Cation‐, and Anion‐ Redox During Electrochemical Cycling of HE‐NCM Cathodes

A driving force for the battery operation, that defines the open circuit voltage of a battery, is the difference in the chemical potentials of lithium (Δµ_Li_) in the cathode (µ_C_^Li^) and in the anode (µ_A_^Li^). Previous model considerations supported by our recent experimental studies have demonstrated that the electronic part (µ_C_^e^) of the chemical potential,^[^ ^32^ ^]^ plays the dominating role in the battery voltage as compared to the ionic part ($\mu_{C}^{\mathrm{Li}+}$).^[^ ^33^, ^34^, ^35^ ^]^ In terms of the evolution of the electronic structure upon electrochemical cycle, charging (discharging) will lead to removal (accommodation) of a Li^+^ ion from (in) the cathode that will immediately lead to removal (accepting) of a valence electron from HE‐NCM to satisfy the charge‐neutrality principle. Accordingly, the *E*_F_ of the cathode is increased upon charging and decreased upon discharging.^[^ ^3^ ^]^ In other words, the internal work function, ϕ_e_, is increased by delithiation of the cathodes, whereas binding energy of the VBM is decreased versus *E*_F_. The opposite direction takes place upon lithiation of the cathodes. Note, this simplified model does not take into account the surface effects, such as a double layer potential drop (i.e., dipole potential, δ_dip_) at the surface of the cathode material occurred, for example, due to the cathode/electrolyte interfacial (CEI) layer formation. Nevertheless, the *E*_F_ energy shift, coupled to the changes in occupation of the M 3d‐ and O 2p‐ electronic states versus cycling, allows to determine the voltage profiles upon cycling various intercalation battery systems.^[^ ^34^, ^36^ ^]^ Moreover, by tracking the current *E*_F_ position and knowing redox levels of the electrolyte components (and impurities, for instance water), the nature of various chemical reactions occurred at the electrode/electrolyte interface, and which are not associated with delithiation/lithiation of the electrodes, can be explained.^[^ ^31^, ^37^ ^]^ In spite of the importance of band structure analysis for advanced batteries, still only few activities are devoted to its practical application for the battery field.^[^ ^31^, ^35^, ^38^ ^]^ Here, we demonstrate the experimental validity of the electrochemical potential evolution upon delithiation (lithiation) of the HE‐NCM cathodes (Figure 3c and Table 1; Figure S10, Supporting Information).

**Table 1:** Evolution of the work function (ϕ_e‐_^T^ and ϕ_e‐_^U^), valence band maximum (VBM) and ionization potential (IP^T^ and IP^U^) of the *treated*‐ and *untreated*‐ *HE‐NCM* cathodes.

*Treated HE‐NCM*	ϕ_e‐_^T^ [eV](hv = 47 eV)	VBM [eV](hv = 250 eV)	IP^T^ [eV](VBM + ϕ_e‐_^T^)	VBM [eV](hv = 1486.7 eV)
As‐prepared	4.70 ± 0.1	0.75 ± 0.1	5.45 ± 0.2	1.1 ± 0.1
3.7 V charge	5.05 ± 0.1	0.60 eV ± 0.1	5.65 ± 0.2	0.8 ± 0.1
4.5 V charge	5.25 ± 0.1	0.50 ± 0.1	5.75 ± 0.2	0.4 ± 0.1
4.6 V charge	5.26 ± 0.1	0.22 ± 0.1	5.48 ± 0.2	0.3 ± 0.1
4.8 V charge	5.37 ± 0.1	0.18 ± 0.1	5.55 eV ± 0.2	0.4 ± 0.1
5.1 V charge	≈ 5.43^a^ ^)^	≈ 0.15^a^ ^)^	5.58	–
*Untreated* *HE‐NCM*	ϕ_e‐_^U^ (hv = 90 eV)	VBM (hv = 450 eV)	IP^U^ eV (VBM + ϕ_e‐_^U^)	VBM^c)^ (hv = 1486.7 eV)
As‐prepared	4.45 ± 0.1 eV^b)^	0.72 ± 0.1 eV	5.17 ± 0.2 eV	0.85 ± 0.1 eV
4.5 V charge	5.20 ± 0.1 eV	0.55 ± 0.1 eV	5.75 ± 0.2 eV	0.50 ± 0.1 eV
4.6 V charge	5.25 ± 0.1 eV	0.35 ± 0.1 eV	5.60 ± 0.2 eV	–
4.8 V charge	5.77 ± 0.1	0.15 ± 0.1 eV	5.90 ± 0.1 eV	0.15 ± 0.1 eV

^a)^ The values obtained by a linear extrapolation of ϕ_e‐_^T^ and VBM to 5.1 V;

^b)^ ϕ_e‐_^U^ was measured at hv = 47 eV;

^c)^ VBM was measured on the *untreated HE‐NCM* (without conductive carbon and PVDF). The work function is defined as *E*_F_ with respect to the vacuum level, *E*_vac_. The ionization potential, IP = ϕ_e‐_ + (*E*_F_ − VBM) + Δδ_dip_, is defined as the energy between the highest occupied state (i.e., VBM) and *E*_vac_. Some discrepancies in the equal change of the work function, ϕ_e‐_, and VBM are caused by a surface dipole layer, Δδ_dip_, which depends on the interfacial conditions (e.g., cathode electrolyte interface (CEI) layer formation). The downward band bending in the as‐prepared *treated‐* and *untreated‐* cathodes is evidenced by a shift of VBM to higher binding energy (i.e., away from *E*_F_) at higher photon energies, which probe the electronic structure deeper into the bulk.

#### 2.3.1 Cationic Redox

**Figure 4:**
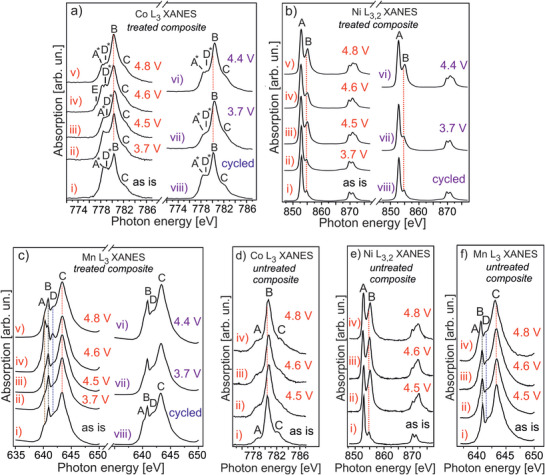
Evolution of Co L_3_, Ni L_3,2_ and Mn L_3_ XANES of the *treated‐* (a–c) and *untreated‐* (d–f) *HE‐NCM* cathodes versus charging (a–f) and discharging (a–c) during the first electrochemical cycle with the upper cut‐off of 4.8 V. As‐prepared are labelled by “as is” (i), the charging states are colored in red, the discharging states are colored in violet [a–c(vi–vii)], cycled [a–c(viii)]. a) Co L_3_ XANES: a steadily intensity decrease of the low energy A^*^ shoulder by charging the *treated HE‐NCM* up to 4.5 V [a(i–iii)] evidences oxidation of Co^2+^; the A^*^, E and D^*^ features inherent to Co^2+^ state increase in intensity upon charging the *treated HE‐NCM* to 4.6 V [a(iv)]; a shift of the B to higher energies upon charging the cathodes [a(i–v), d] evidences an increase in the oxidation state of Co. The opposite B shift occurs upon discharging the cathodes [a(vi–viii)]. b, e) Ni L_3,2_ XANES: the intensity increase of the B shoulder and its shift to higher energies are due to an increase in the oxidation state of Ni. Upon discharging, the B decreased in the intensity and shifted in the opposite direction, thereby confirming reduction to the Ni^2+^ state in the *treated HE‐NCM*. c,f) Mn L_3_ XANES: the increase in intensity of the A (≈ 640.2 eV), B (640.8 eV), and D (641.8 eV) shoulders of the *treated HE‐NCM* cathode [c(iv)] is due to the contribution of Mn^2+^ and Mn^3+^ (see calculations in Figure 1e); Mn L_3_ XANES is not changed markedly by charging the *untreated HE‐NCM* cathode (f).

**Figure 5:**
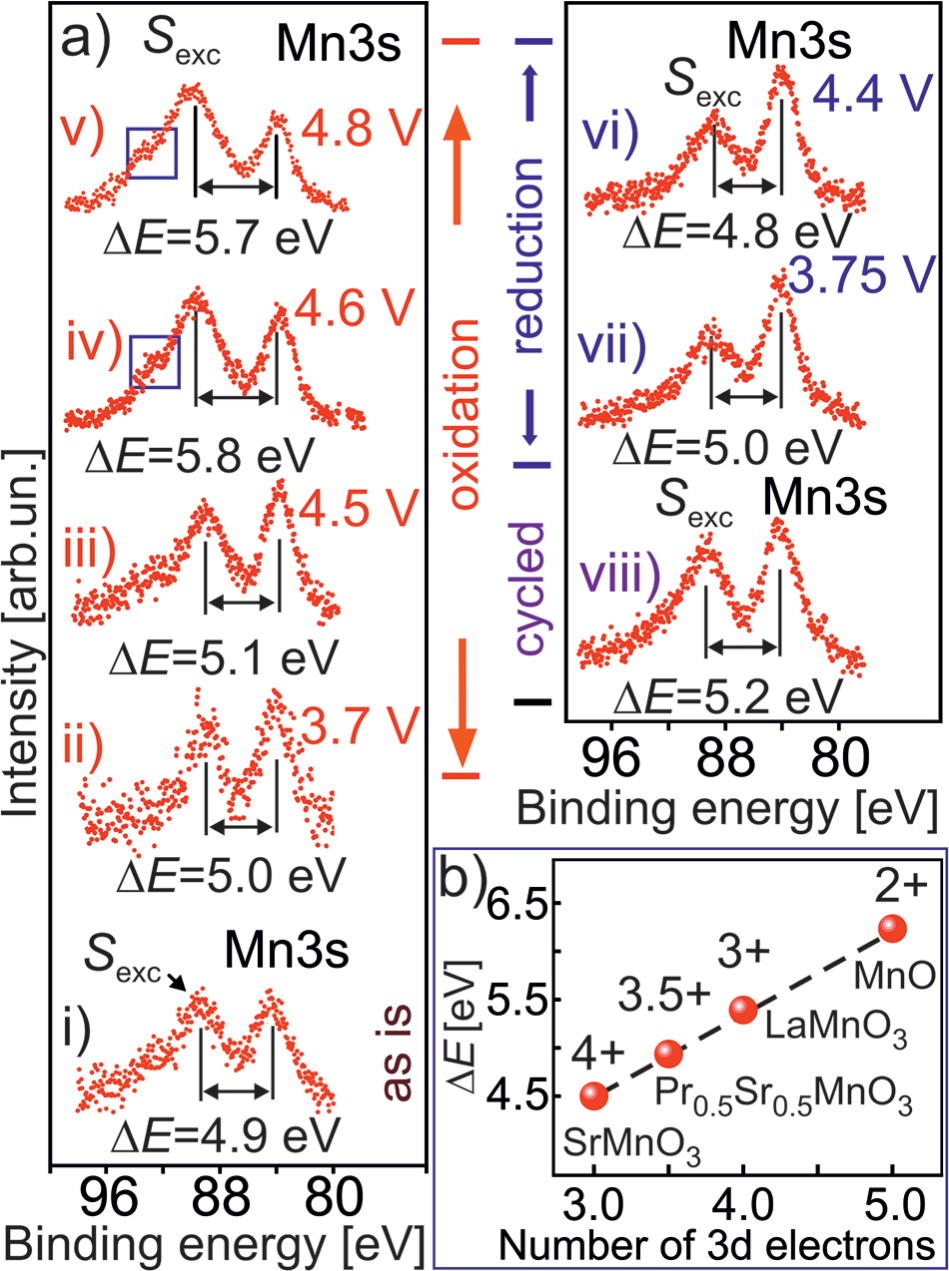
a) Evolution of the Mn 3s photoelectron spectra (hν = 1486.7 eV) of the *treated HE‐NCM* cathode versus charging and discharging during the first electrochemical cycle with the upper cut‐off of 4.8 V. As‐prepared sample (i), the charged states of 3.7 V (ii), 4.5 V (iii), 4.6 V (iv), 4.8 V (v), discharged states of 4.4 V (vi) and 3.75 V (vii), cycled (viii). Δ*E* is the exchange splitting defined as the energy difference between the Mn 3s peak and exchange satellite, *S*_exc_. A higher number of electrons on the Mn 3d level (i.e., higher reduction of Mn) leads to a stronger Δ*E*. The increase in the *S*_exc_ intensity with respect to Mn 3s is assigned to the impurities (the binding energy area is labelled by the blue box).^[^ ^21^ ^]^ b) The dependence of Δ*E* versus number of 3d electrons for various manganese oxides.^[^ ^21^, ^39^ ^]^

**Figure 6:**
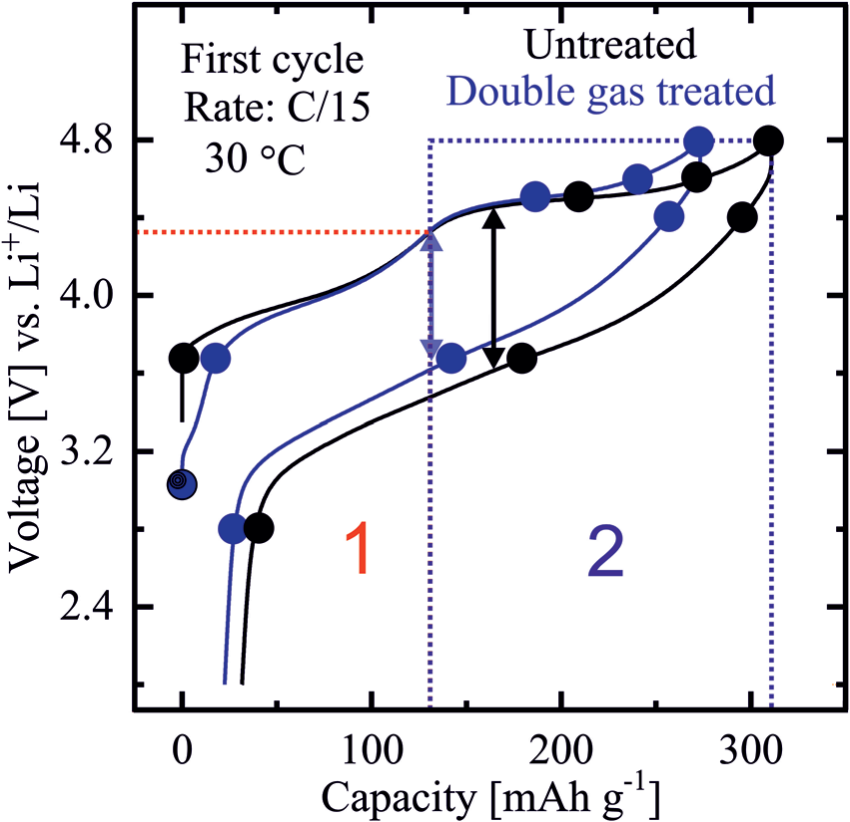
Voltage profiles of the first charge‐discharge cycle of the *untreated* (black) and the double gas (SO_2_ and NH_3_) *treated* (blue) *HE‐NCM* cathodes at C/15 rate in 1 M LiPF_6_ /EC‐EMC solution. Voltage hysteresis is shown by arrows. The charging/discharging potentials, upon which the electronic properties of the cathodes were probed by XPS, SPES, XANES, are labeled by solid filled circles. The plateau regions labeled by 1 and 2 correspond to oxidation of the cations and anion, respectively.

Based on the energy band diagrams derived from our SPES, XANES and RPES experiments (Figure 3a,b), as well as in agreement with theoretical expectations, *E*_F_ enters the occupied Co 3d‐ and Ni 3d‐ states at first in the beginning of charging the HE‐NCM cathodes. In turn, this leads to the oxidation of Co and Ni as supported by the Co L_3_‐ and Ni L_3_‐XANES, whose spectral features change in intensity (**Figure** **4**a,b,d,e; Figures S13 and S14, Supporting Information), as well as by the Co K‐ and Ni K‐ XANES which shift to higher energies by charging the cathodes ≤ 4.5 V (Figures S15 and S16, Supporting Information). Mn ions do not change their oxidation state by charging the *treated‐ and untreated‐ HE‐NCM* cathodes below 4.5 V as evidenced by the Mn L XANES and Mn 3s photoelectron spectra (Figures 4c,f and **5**a‐i–iii and **Table** **2**; Figure S17, Supporting Information). Such finding is in good agreement with the DOS distribution of the as‐prepared HE‐NCMs where the Mn 3d states have a higher binding energy with respect to the Co 3d‐ and Ni 3d‐ states (Figure 2a–d; Figure S6, Supporting Information). No significant changes in the O K‐edge XANES up to 3.7 V evidence that lattice oxygen is not involved in the anionic redox process (Figure S18, Supporting Information). The minor changes in the O K XANES of the *treated HE‐NCM* cathode are ascribed to evolution of the oxygen‐related species induced by the NH_3_ surface gas treatment (see discussion in more details in the Discussion Section S3, Supporting Information). Importantly, *E*_F_ does not cross the O 2p state of Li_2_O at a charging state below 4.5 V that is evidenced as the absence of the marked changes in the C peak intensity of the O K XANES (Figure S18, Supporting Information). In the voltage profiles of the HE‐NCM cathodes, the plateau region labelled by 1 corresponds to oxidation of the relevant transition metals (**Figure** **6**). A more detailed discussion on the electrochemical properties of the HE‐NCM cathodes is provided in the Discussion Section S4 and Figure S11 (Supporting Information).

**Table 2:** The binding energy evolution of the Mn 3s photoemission and exchange satellite (*S*_exc_) versus charging/discharging of the *treated HE‐NCM* cathode.

	Mn 3s [eV]	*S*_exc_ [eV]	Δ*E*_Mn3s‐_ *_Sexc_* [eV]	Valence
Pristine	84.25 ± 0.05	89.15 ± 0.05	4.9 ± 0.1	≈ 3.5+
Soaking	83.85 ± 0.05	89.2 ± 0.1	5.35 ± 0.15	≈ 3+
3.7 V charged	84.0	89.0	5.0 ± 0.1	≈ 3.4
4.5 V charged	83.8	88.9 ± 0.1	5.1 ± 0.1	≈ 3.3+
4.6 V charged	83.7	89.5 ± 0.1	5.8 ± 0.1	≈ 2.3+
4.8 V charged	84.0	89.7 ± 0.2	5.7 ± 0.2	≈ 2.4+
4.4 V disch.	84.0 ± 0.1	88.8 ± 0.1	4.8 ± 0.2	≈ 3.6+
3.75 V disch.	84.05 ± 0.05	89.0 ± 0.1	4.95 ± 0.15	≈ 3.4+
After 1^st^ cycle	84.05 ± 0.05	89.25 ± 0.05	5.2 ± 0.1	≈ 3.1

#### 2.3.2 Activation of Anionic Redox

**Figure 7:**
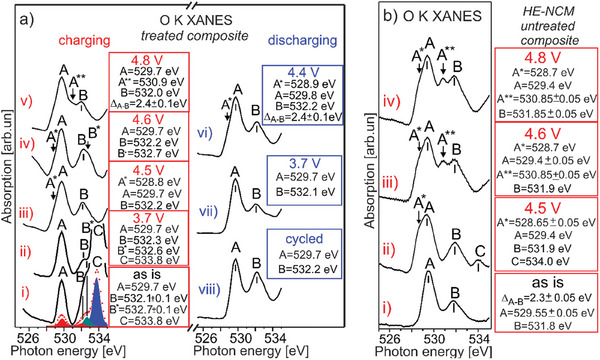
The evolution of O K XANES of the *treated‐* (a) and *untreated‐* (b) *HE‐NCM* cathodes versus charging (a, b), and discharging (a) during the first electrochemical cycle with the upper cut‐off of 4.8 V. The charging states are colored in red, the discharging states are colored in blue [a(vi‐vii)]. a, b) as‐prepared (i), charged to: 3.7 V [a(ii)], 4.5 V [a(iii), b(ii)], 4.6 V [a(iv), b(iii)], 4.8 V [a(v), b(vi)]; discharged to: 4.4 V [a(vi)], 3.7 V [a(vii)], cycled [a(viii)]. The electron removal from the lattice oxygen leads to the O^−^ hole formation (denoted as A^*^) [a(iii)]. A decrease of the empty states in the lattice oxygen (vanishing of the A^*^ intensity) correlates with an increase of the shoulder B^*^ due to a hole transfer to a non‐bonding O 2p orbital [a(iv)]. The B and B^*^ states are assigned to M 3d (e_g_) and non‐bonding O 2p orbital, respectively. b) A^*^, A^**^ and C are empty states due to a hole formation on the lattice oxygen, molecular oxygen (O_2_) and Li_2_O, respectively.

The *E*_F_ approach to the O 2p state of the lattice oxygen hybridized with the M 3d states leads to distinct differences in the O K XANES (Figures S18a(iv),b(ii), and S19, Supporting Information). At the 4.5 V charging state, the *E*_F_ position equals to 5.25 ± 0.1 eV (versus the vacuum level, *E*_vac_) for both the *untreated*‐ and *treated*‐ *HE‐NCM* cathodes (Table 1). For the *treated HE‐NCM*, this means that *E*_F_ is equal to the energy of the non‐bonding O 2p orbital of Li_2_O, which lies in the ≈ 4.5 − ≈ 5.5 eV binding energy range (Figure 2a). As the result, the C peak (the empty states of a Li−O−Li bond) disappears irreversibly in the O K XANES (**Figure** **7**a‐iii), thereby evidencing oxidation of the Li_2_O related non‐bonding states and removal of lithium oxide from HE‐NCM. In addition, the A^*^ shoulder (528.8 eV) occurs in O K XANES associated with the lattice oxygen in both the *treated*‐ and *untreated*‐ *HE‐NCM* cathodes (Figure 7a‐iii,b‐ii). Whilst there are different models of oxidation mechanisms in LRC, namely O^2−^/O^−^ redox, or O^2−^/(O_2_)^n−^ (peroxo‐like dimer species) or isolated O^−^ anions,^[^ ^40^ ^]^ we assign the A^*^ feature with the hole formation on oxygen (lattice) in agreement with the previous work.^[^ ^31^ ^]^ Note that peroxide formation is not detected also by Raman spectroscopy (Figure S20, Supporting Information). Thus, the movement of *E*_F_ into the O 2p state of the lattice oxygen leads to the O^2−^ oxidation, or in other words, to hole transfer to the O 2p state. In the voltage profiles, the plateau region associated with oxygen redox is labelled by 2 (Figure 6). An enhanced holes formation (A^*^ = 528.8 eV) in the *untreated HE‐NCM* evidences a stronger distortion of the oxygen site as compared to the *treated HE‐NCM* (Figure 7a‐iii,b‐ii). A higher amount of oxygen holes in *untreated HE‐NCM* is accompanied with Li_2_O activation (irreversible) manifested by the C peak at 534.0 eV (Figure 7b‐ii), which evidences te triggering oxidation of the lattice oxygen. In this regard, *untreated HE‐NCM* behaves as a classical LRC, where charging the cathodes above 4.4 V leads to activation of Li_2_MnO_3_ via the formation of Li_2_O (supported also by operando Raman spectroscopy, Figure S20, Supporting Information), followed by its decomposition to evolve oxygen gas.^[^ ^9^ ^]^ In parallel to the involvement of the lattice oxygen in charge compensation, the transition metals are more oxidized in both cathodes. This is evidenced by changes in the Co L‐, Co K‐, Ni L‐, and Ni K‐ XANES (Figure 4a‐iii,b‐iii,d‐ii,e‐iii; Figures S13−S16, Supporting Information), as well as in a relative intensity increase of the O K XANES peaks associated with the t_2__g_ and e_g_ empty states mostly of Co and Ni (Figure S19‐iii, Supporting Information). Minor changes in the shape of the Mn L XANES, as well as the Mn 3s photoelectron spectra (Figure S17a‐iii,b‐ii, Supporting Information; Figure 5a‐iv) support inactivity of Mn 3d in charge compensation at such voltage. Interestingly to note that a higher oxidation of the Ni ions in the *untreated HE‐NCM*, as compared to the treated counterpart (see Ni L_3,2_ XANES in Figure 4b‐iii,e‐ii), correlates with a higher distortion of the lattice oxygen in the *untreated HE‐NCM* charged to the same potential (4.5 V) (Figure 7a‐iii,b‐ii). As such, we can assume that *E*_F_ penetrates deeper into the Ni 3d‐ and O 2p‐ occupied states of the *untreated HE‐NCM*. This suggestion agrees with the relative energies of the electronic states of as‐prepared cathodes, where *E*_F_ is situated closer to the VBM of the *untreated HE‐NCM* as illustrated by the energy diagrams (Figure 3a,b).

By charging the HE‐NCM cathodes to a higher potential (4.6 V), the *E*_F_ shifts deeper down (Figure 3c and Table 1). Therefore, more Co and Ni ions are expected to be involved in the charge compensation, thereby getting a higher oxidation to the Co^4+^ and Ni^3+^/Ni^4+^ states, as generally accepted in the conventional NCM layered oxides. Instead, Co and Ni are not further oxidized in the *untreated HE‐NCM composite* (Figure 4d‐iii,e‐iii; Figures S14b‐iii, S15c‐iii, and S16c‐iii,d‐iii, Supporting Information). The trend in oxidation of transition metals agrees with the ligand‐to‐metal charge transfer (LMCT) mechanism inherent for anionic redox LCR.^[^ ^41^ ^]^ We suggest that the charge extracted from M 3d is balanced back by charge transfer from O 2p state according to Co^4+^ – O^2−^ → Co^3+σ^– O^n^^−^ and Ni^3+/4+^ – O^2−^ → Ni^2+σ^ – O^n−^ (0 ≤ σ < 1; 1 < n < 2). In the *untreated HE‐NCM composite*, such electronic charge transfer is accompanied with a strong distortion at the oxygen site and the molecular oxygen (O_2_) formation (A^**^≈ 530.9 eV,^[^ ^23^ ^]^) in addition to the oxygen holes (A^*^ = 528.7 eV) (Figure 7b‐iii). It was recently pointed out that generation of O_2_ triggers the formation of closed voids/cracks near the particles of the active material.^[^ ^42^ ^]^ Such voids trap molecular oxygen during cycling that limits the involvement of O_2_ in electrochemical activity,^[^ ^42^ ^]^ and results in an increasing voltage fade.

Whilst our finding reveals that the M ions are no further oxidized in the *untreated* cathode being charged to 4.6 V, they are obviously reduced in the *treated* cathode in comparison with charging state of 4.5 V (see the Co L‐ and Ni L‐ XANES in Figure 4a‐iv,b‐iv). A more pronounced reduction occurs at the Mn‐ site with the distinct contribution of Mn^2+^/Mn^3+^ in Mn L XANES (see Figure 4c‐iv; Figure S17a‐iv, Supporting Information, in comparison with theoretical calculations, Figure 1e). A nominal oxidation state of Mn is 2.3+ at 4.6 V charging state, as determined from Mn 3s photoelectron emission (Figure 5a‐v and **Table** **2**). Reduction of transition metals at the cathode/electrolyte interface is also evidenced by a significant increase of occupation of the O 2p (lattice) vacancies (Figure S18v, Supporting Information). Such a reduction is often assigned with M‐dissolution, side reactions, or electrolyte‐to‐surface electronic charge transfer, i.e., electrolyte oxidation.^[^ ^31^, ^43^ ^]^ However, our results show that the electrochemical potential of both cathodes has very similar values, whereas the ionization potential of the *treated* cathode is even lower as compared to its *untreated* counterpart (Table 1). Such a finding rules out the reduction of the transition metals at the surface due to an electron transfer from the electronic levels of electrolyte to the M 3d levels of the *treated* cathode, taking into account that the same electrolyte is used for both HE‐NCM cathodes. A stronger reduction of the M‐ions in the *treated* cathode at 4.6 V cannot be also explained by a deeper penetration of *E*_F_ into the M 3d and O 2p (lattice) levels. This would lead to a stronger involvement of the lattice oxygen in the charge compensation, therefore, to the formation of greater number of holes at the oxygen site. In contrast to that, we observe an essential decrease of the number of O^n^^−^ holes (A^*^ = 528.8 eV) in the *treated* cathode (Figure 7a‐iv). Importantly, the oxidation of O^2−^ ions (lattice oxygen) does not form O_2_ by charging the *treated HE‐NCM* *composite* to 4.6 V that agrees with a decrease in voltage hysteresis (Figure 6).

Mitigation of distortion of the oxygen site correlates with the occurrence of the shoulder B^*^≈ 532.8 eV in the O K XANES, which is missing in the *untreated HE‐NCM* cathode (Figure 7a‐iv,b‐iii). The revealed A‐B^*^ difference of 3.1 V (Figure S19‐iv, Supporting Information) is higher than the crystal field splitting for both the *O*_h_ and *T*_d_ symmetries. This makes assigning of the B^*^ shoulder to an empty 3d state unlikely, at least for the considered environments with oxygen (see more discussion Section S5, Supporting Information).

Therefore, it is reasonable to assume that the presence of the O 2p empty states (assumed non‐bonding) in addition to the O 2p (lattice) and overlapping these oxygen states with the M 3d states, might cause a complex charge transfer between the O 2p‐ and M 3d‐ states upon charging the *treated HE‐NCM* cathode. As an example, the charge extracted from M 3d orbital is balanced back from one of O 2p states to the e_g_ or t_2g_ orbitals, depending on the electronic configuration of M‐ion in a charged state of HE‐NCM (see Figure S1a, Supporting Information). The relevant occupation of the O 2p (lattice) vacancies in the *treated* cathode, which were formed by charging to 4.5 V, mitigates the distortion of the lattice oxygen and the oxygen loss in comparison with the *untreated* cathode. As for lithium peroxide (Li_2_O_2_), we cannot support its existence in both *treated*‐ and *untreated‐* cathodes due to the absence of the Raman peak at ≈ 790 cm^−1^ (Figure S20, Supporting Information) and an inability to resolve peroxide in O K XANES due to its energy vicinity to O_2_ (the former is commonly observed at ≈ 530.5 eV of the O K‐edge,^[^ ^44^ ^]^).

#### 2.3.3 A Deep Stage of Delithiation

**Figure 8:**
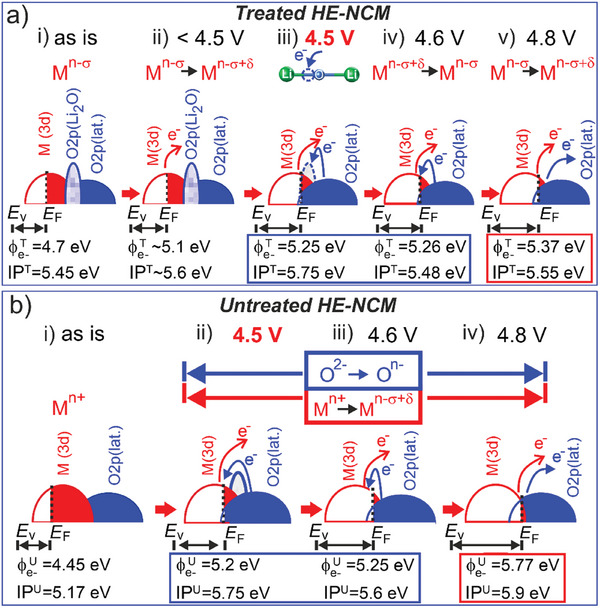
Evolution of the electrochemical potential and oxidation states of the cations and the anion upon delithiation during the first charge of the *treated‐* (a) and *untreated‐* (b) *HE‐NCM* cathodes. a(i), b(i) The *E*_F_ position in as‐prepared cathodes; a‐ii) oxidation of the M cations (δ < 1 and δ < 2 for Co and Ni, respectively) below 4.5 V; (iii) an illustration of the possible electron transfer to vacancy in the Li−O−Li bond via oxidation of the lattice oxygen (O^2−^ → O^n−^: 1 *<* n *<* 2) at 4.5 V; (iv) oxidation of Co and Ni due to the *E*_F_ shift and a partial reduction (labeled by σ) of Co, Ni, and Mn via an electron transfer from the O 2p (lattice) to the M 3d levels at 4.6 V; (v) oxidation of M 3d states and an electron removal from the lattice oxygen at 4.8 V. The O 2p state of N─O bond is not shown. b) (ii, iii) oxidation of Co and Ni due to the *E*_F_ shift, and a partial reduction of Co, Ni, and Mn via an electron transfer from the O 2p (lattice) to the M 3d levels in the 4.5 – 4.6 V range. The O 2p state of Li_2_O activated at 4.5 V is also shown (ii); (iv) oxidation of M 3d states and an electron removal from the lattice oxygen at 4.8 V. The empty‐ and occupied‐ states are shown by line and area plots, respectively. ϕ_e‐_^T^ (ϕ_e‐_^U^), IP^T^ (IP^U^), *E*_F,_ and *E*_v_ are the work function, ionization potential, Fermi level, and vacuum level, respectively. The ionization potentials were estimated at the VBM and ϕ_e‐_^T^ (ϕ^U^_e‐_) measured at hν = 250 eV and hν = 47 eV, respectively (Table 1). The decrease of IP^T^ and IP^U^, whereas ϕ_e‐_^T^ and ϕ_e‐_^U^ remained almost unchanged [a(iii, iv), b(ii, iii)] evidences a double layer potential drop, Δδ_dip_, formed at the cathode/electrolyte interface. Δδ_dip_ in fact lowers the work function.

Upon an increasing the charging potential to 4.8 V, *E*_F_ moves deeper into the M 3d hybridized with O 2p (lattice) states manifested by a higher values of the work function (Table 1). In the *treated HE‐NCM* cathode, the number of empty M 3d states increases again evidencing further oxidation of the transition metals. This conclusion is supported by an intensity increase of the A and B peaks in the O K XANES (Figure S19, Supporting Information) and the change in the shape of the M L‐edges XANES (Figure 4c‐v; Figures S13‐v and S14a‐v, Supporting Information). In the *untreated HE‐NCM* cathode, the M ions are no further oxidized (Figure 4d‐iv,f‐iv), whereas a higher amount of O^n^^−^ holes (lattice) and O_2_ species are formed in comparison to the status at 4.5 V (Figure 7b‐iv). Importantly, the highest oxidation states, such as Co^4+^ and Ni^4+^, are not attained in either cathode, thereby ruling out a complete oxidation of the transition metals at 4.8 V (see more discussion on oxidation states of the M ions in Discussion Section S6, Supporting Information). Thus, this partial oxidation of the transition metals in HE‐NCM cathodes agrees well with the LMTC model,^[^ ^41^ ^]^ according to which the Co^4+^ and Ni^4+^ formal oxidation states are compensated by an electron transfer from O^2−^ 2p states. Note that even for conventional layered structure oxides, a strong hybridization of O 2p‐ and M 3d‐ states leads to a competition between cationic and anionic redox processes at a deep stage of delithiation.^[^ ^45^ ^]^ Importantly, the O K XANES of the *treated HE‐NCM* cathode shows only a minor distortion of the lattice oxygen at 4.8 V, very similar to that occurring at 4.5 V (Figure 7a‐iii,v). The difference is a slightly visible shoulder at A^**^ = 530.9 eV occurred at the higher charging potential that evidences molecular oxygen (O_2_) formation at 4.8 V (Figure 7a‐v). This finding indicates a higher stability threshold for the lattice oxygen of the *treated HE‐NCM* cathode. The difference in the electronic charge transfer mechanisms upon delithiation of the *treated*‐ and *untreated*‐ *HE‐NCM* cathodes is illustrated (**Figure** **8**).

### 2.4 Effect of the Electronic Structure Design and Surface Coating on Stability of High‐Voltage Cathode Materials at Charging State Above 4.8 V

**Figure 9:**
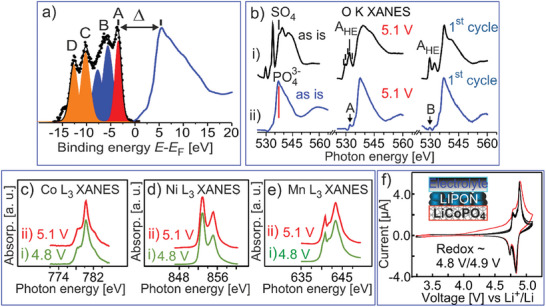
a) The electronic configuration of LCP olivine‐ structure thin film cathode (without both conductive carbon and binder) near the *E*_F_ = 0 derived from photoemission experiments: on the left, the valence band (VB) structure (hv = 1486.7 eV); the right, O K‐edge XANES (TEY). The spectra are plotted on a unified energy scale. A = 3.7 eV is associated with the t_2__g_ and e_g_ states of Co, B = 6.0 eV is ascribed to the Co 3d hybridized with O 2p bonding and antibonding states, the C = 10.2 eV and D = 12.6 eV are related to the oxygen and phosphorous strongly bound states, respectively. Δ = 9.3 eV is the energy gap between the Co 3d and ${\mathrm{PO}}_{4}^{3-}$. In the O K‐edge XANES the most intense peak situated at ≈ 5 eV from the *E*_F_ is occurred due to the electron transition from the O 1s core‐level to the empty O 2p band. The broadness of the O K‐ edge of phosphates, as compared to layered structure oxides, reflects a strong covalent oxygen−phosphor bonding in the ${\mathrm{PO}}_{4}^{3-}$ unit.^[^ ^35^ ^]^ b) The evolution of O K‐edge XANES of the *treated HE‐NCM* cathode (i) and LCP thin film cathode (ii) by charging to 5.1 V (in the middle) and the first electrochemical cycle with the upper cut‐off of 5.1 V (on the right). The assignment of the A_HE_ (i), A (ii) and B (ii) is described (Figures S21a and S24, Supporting Information). c–e) Co L_3_ (c), Ni L_3_ (d), Mn L_3_ (e) XANES of the *treated HE‐NCM* cathode charged to 4.8 V (i) and 5.1 V (ii) during the first cycle. f) Cyclic voltamograms of the LCP thin‐film cathode coated with LiPON charged to 5.1 V. The CV after the first electrochemical cycle is shown in red. All potentials are referenced versus Li^+^/Li.

Getting higher energy density via a complete delithiation of the cathode materials is attractive, although mitigating the O_2_ lattice release coupled to the structural instability upon a deep charging (> 4.8 V vs Li^+^/Li) is a challenge. Considering the intrinsic stability of the high‐voltage cathodes, the key question is how far the O 2p states (lattice) can be engineered below the Fermi level to prevent their involvement in charge compensation. A good example is high‐ voltage LiMPO_4_ (M = Co, Ni) olivine structure cathodes with the more ionic character of the chemical bonding between M 3d and O 2p states provided by the inductive effect.^[^ ^46^ ^]^ A more electronegative P^5+^ countercation pulls the electronic density away from the M 3d state, thereby resulting in enlarging the M 3d – O 2p bond distance and reducing the M–O covalency that leads to M^2+/3+^ redox couple above 4.8 V. As the result, the M 3d–O 2p states are sharp and well separated from each other, as probed by photoelectron spectroscopy (**Figure** **9**a; see more discussion on the electronic structure of LiCoPO_4_ in Discussion Section S7, Supporting Information). Due to a large energy separation between the M 3d and O2p levels, the *E*_F_ moves down upon charging the olivine‐type cathodes without pinning the O2p state even at high charging potentials.^[^ ^35^ ^]^

The O K XANES of the *treated HE‐NCM* cathode and LiCoPO_4_ thin‐film cathode cycled to 5.1 V are shown in Figure 9b. The several chemical states (denoted as A_HE_) arise in the *treated HE‐NCM* cathode at the highest charging potential (see O K XANES in more details in Figure S21a, Supporting Information). The shoulder at 528.8 eV and the next peak (529.7 eV) are ascribed to the O^−^ species and M 3d−O 2p hybridized states, respectively, in agreement with our finding for HE‐NCM charged to 4.8 V (Figure 7a‐v). The shoulder at 531.1 eV is mostly ascribed to molecular oxygen (O_2_) formation. In this regard, the spectral features in the O K XANES are very similar to those observed for the *untreated HE‐NCM* cathode charged at 4.8 V (Figure 7b‐iv). The main difference is an unexpectedly high intensity of the M 3d (e_g_) state (532.0 eV) and the occurrence of shoulder at ≈ 533 eV. The revealed changes in O K XANES are mostly associated with the formation of carbonyl‐ (C = O, 531.2 eV), carboxyl‐ (O−C = O, 532 eV) and carboxylic‐ (533 eV) groups,^[^ ^47^ ^]^ as well as Li_2_CO_2_ (533 eV,^[^ ^48^ ^]^) due to oxidation of the electrolyte. This conclusion is supported by examination of *E*_F_ and IP of HE‐NCMs (Table 1). The oxidation potentials of a mixture of EC‐EMC carbonate solvent are 4.8 – 4.9 V (vs Li^+^/Li),^[^ ^49^ ^]^ whereas the *E*_F_ and the IP equal to ≈ 5.4 and ≈ 5.6 eV, respectively, when the *treated HE‐NCM* cathode is charged to 5.1 V (Figure 3c and Table 1). This means the *E*_F_ is shifted below the electronic levels of the carbonate solvents that leads to a hole transfer from the cathode to the electrolyte (see the relevant discussion.^[^ ^31^ ^]^). However, such a charge transfer is not accompanied by reduction of the transition metals at the surface in comparison with 4.8 V (Figure 9c,d). Moreover, Ni is even slightly oxidized, although oxidation state of Ni remains lower than 4+ (compare Figure 9d with Figure S22, Supporting Information). The reduction takes place probably at the lattice oxygen, although drawing an explicit conclusion is difficult due to a complexity of the LMCT mechanism at the cathode/electrolyte interface. Nevertheless, the *treated HE‐NCM* cathode demonstrates a good reversibility of the electronic structure after the first electrochemical cycle (Figure 9b‐i,c,d). This finding suggests that the practical capacity of the HE‐NCM cathodes can be further increased by selecting an appropriating electrolyte stable at 5.1 V, although a long‐time cycling stability of the lattice oxygen at such a high potential remains uncertain.

The O K XANES of the LCP thin film cathode shows the lattice oxygen is not involved in redox process at 5.1 V (Figure 9b‐ii, middle). The changes in O K XANES caused by delithiation (Figure S24, Supporting Information) are mostly associated with carboxyl groups due to the electrolyte oxidation (A ≈ 532 eV), as well as alteration in the Co 3d−O 2p hybridization and an increasing number of unoccupied Co 3d−O 2p hybridized states (B ≈ 529.9 eV).^[^ ^35^ ^]^ Thus, enough distance of the O 2p band from *E*_F_ in LCP excludes the involvement of the lattice oxygen in charge compensation at 5.1 V. Nevertheless, remaining of the B peak after cycling (Figure 9b‐ii), is a sign of an irreversible occupation of the Co 3d empty states. The issue with an instability of the electrolyte at the surface of the cathode materials in a deep charging state can be solved via design of the interfacial electronic properties. Besides the SO_2_ and NH_3_ gas treatment reported here for HE‐NCM, coating of the redox active oxides also changes their electrochemical potential with respect to the electronic levels of electrolyte, as shown in our recent research.^[^ ^50^ ^]^ If so, the material for an efficient coating can be selected based on the energy diagrams and electrochemical potential of the redox active oxides obtained from the experiments.^[^ ^50^ ^]^ Based on such a forecast, coating the LCP thin‐film cathodes with lithium phosphorus oxynitride (LiPON) results in a significant improvement in cycling stability (Figure 9f; Figure S25, Supporting Information).^[^ ^51^ ^]^ Further discussion on the selection criteria of the artificial interfaces is provided in the Discussion Section S8 (Supporting Information) (see also ref. [50]). We note that severe issues associated with dissolution of transition metals at the electrode/electrolyte interface of high‐voltage layered structure cathodes, as well as gas evolution upon charging to a high potential can be overcome by choosing an appropriate coating and by selecting an electrolyte with a high electrochemical stability window, respectively, ensuring that the cathode and electrolyte are chemically compatible. In this regard, the modified surface of the *treated* HE‐NCM cathode plays also a role for the protective layer, namely, the treatment modifies the *E*_F_ position favorable to avoid the involvement of lattice oxygen into anionic redox at a high charging potential, thereby improving cyclic stability. The above‐described approaches can be used to design artificial interfaces for various electrochemical systems, in particularly by means of the electrode material´s gas treatment, as it was previously reported.^[^ ^20^, ^52^, ^53^ ^]^

## 3 Conclusion

Here, the energy band diagrams concept is introduced to explore the intrinsic voltage limit of high‐voltage cathodes and to explain the evolution of the cationic and anionic redox upon electrochemical cycling up to ≤ 5.1 V versus Li^+^/Li. This approach, coupled to examination of the changes in the electrochemical potential and ionization potential, enables to elucidate the mechanisms of chemical reactions occurred at the cathode/electrolyte interface, which differ from redox processes associated with (de‐)lithiation reactions. The general remarks on the separation of lithiation/delithiation reactions from interfacial chemical reactions can be found in the Discussion Section S9 (Supporting Information). Specifically, we exam the effect of SO_2_ and NH_3_ double gas treatment of *x*Li_2_MnO_3_·(1−*x*)Li(M)O_2_ (HE‐NCM) layered structure cathodes using advanced electron spectroscopy techniques combined with theoretical calculations and operando Raman experiments. The SO_2_ and NH_3_ treatment alter the surface chemistry and electronic structure via a partial reduction of Co and Mn ions and the formation of Li_2_O with the O 2p orbital situated between the hybridized M 3d and O 2p (lattice) levels. The surface modification lifts up the electrochemical potential of the *treated* cathode toward the unoccupied M 3d and O 2p (lattice) electronic states. This shift in the *E*_F_ position is beneficial for the stability of the cathode, because upon delithiation, the Fermi level is shifted downwards into the occupied O 2p states to a less extent, as compared to the untreated counterpart charged to the same potential (Figure 8a(v),8b(iv)). As the result, the *treated HE‐NCM* cathode exhibits reduced oxygen loss, voltage hysteresis and interfacial impedance. In the as‐prepared *untreated HE‐NCM* cathode, the existence of Li_2_O is not confirmed neither at the surface nor in the bulk. This finding means that the relevant Li−O−Li bond (if any) is structurally integrated into *x*Li_2_MnO_3_·(1−*x*)Li(M)O_2_ and, therefore, cannot be resolved as a separated phase by using the independent experimental techniques. However, Li_2_O is activated by charging the *untreated HE‐NCM* cathode to 4.5 V, in agreement with a generally accepted model of anionic redox in *x*Li_2_MnO_3_·(1−*x*)Li(M)O_2_.^[^ ^9^ ^]^ By comparing the *E*_F_ and IP positions with respect to oxidation levels of the electrolyte components, we are able to separate the lattice distortion of HE‐NCM from the changes in the interfacial composition induced by electrolyte oxidation which occurs by charging the cathodes to 5.1 V. The apparently stable redox activity of the surface layer even at high potentials implies a high stability which in consequence protect the HE‐NCM against oxygen lost. The *treated HE‐NCM* cathodes with available ${\mathrm{SO}}_{4}^{-2}$ bonds at the surface might induce a similar effect in the improvement of the battery performance that incorporation of XO_4_ (X = P, S, Si) units into the bulk of the oxide lattice recently discovered for the Li‐excess integrated rocksalt‐polyanion cathodes.^[^ ^12^ ^]^ The bulk incorporation of polyanion groups mitigates oxygen loss from the lattice, thereby enhancing the cyclic stability.^[^ ^54^ ^]^ The energy band diagram approach, coupled to the prediction of *E*_F_ position upon charging the cathodes to a high voltage, is used to design a protective interface for the LiCoPO_4_ (LCP) olivine structure cathode which commonly degrades fast upon electrochemical cycling. The LCP coated with lithium phosphorous oxynitride (LIPON) shows significant improvements in cycling stability. To sum up, the proposed concept allows us to predict the intrinsic voltage limit of various cathodes,^[^ ^31^, ^55^ ^]^ the interfacial stability upon charging batteries to a high voltages,^[^ ^35^ ^]^ explain the reason of chemical compatibility (or incompatibility) between artificial layers and the electrodes,^[^ ^50^ ^]^ and an efficiency of the redox active oxides with respect to the oxidation of water.^[^ ^56^ ^]^ Conceptually, our research opens new horizons in the interface engineering of high voltage cathodes, thereby enabling to increase the energy density of lithium‐ion batteries. We believe that the proposed approach is reliable and applicable for various cathode materials, such as spinel structure oxides, disordered rocksalt (DRX) cathodes, high entropy layered oxides, etc.

## 4 Experimental Section

### Materials

Details of chemical synthesis of 0.33Li_2_MnO_3_·0.67LiNi_0.4_Co_0.2_Mn_0.4_O_2_ (HE‐NCM) cathode materials were reported elsewhere.^[^ ^19^ ^]^ In this work, the following terminology was used: a) the *untreated HE‐NCM* (pristine, without conductive carbon and PVDF additives), *untreated composite HE‐NCM* = *untreated* cathode (includes conductive carbon and PVDF additives), *treated HE‐NCM* (double‐gas treated with SO_2_ and NH_3_, without conductive carbon and PVDF additives) and *treated composite* = *treated* cathode (double‐gas treated with SO_2_ and NH_3_, includes conductive carbon and PVDF additives). Polyvinylidene difluoride (PVDF) was a binder. The *untreated*‐ and *treated*‐ HE‐NCM powders (without conducting carbon and PVDF) were pressed into Al‐mesh of 7 mm diameter. The LiCoO_2_ (LCO), LiNi_0.2_Co_0.7_Mn_0.1_O_2_ (NCM), and LiCoPO_4_ (LCP) thin film cathode materials were prepared in DAISY–BAT laboratory by radio‐frequency (RF) magnetron sputtering technique.^[^ ^57^ ^]^ The thin‐film deposition processes were described in more details.^[^ ^31^, ^50^, ^55^ ^]^ The conditions for coating of the LCP thin films with phosphorous oxynitride (LIPON) were described elsewhere.^[^ ^50^ ^]^ The LCP thin films and LiPON layer of ≈ 150 and 50 nm thick, respectively, were studied in the current work. After preparation, HE‐NCM materials were stored in a glove box under argon atmosphere (H_2_O and O_2_ contents were <0.5 ppm).^[^ ^21^ ^]^

### Electrochemical Characterization

For the *quasi‐in‐situ* electron spectroscopy experiments, the Swagelok‐type battery cells were assembled in a glovebox. Metallic lithium anode and Celgard2500 as the separator were used. 1 m LiPF_6_ ethylene carbonate (EC): diethyl carbonate (DEC) (50:50) electrolyte (Sigma Aldrich, Germany), and 1 m LiPF_6_ in 4:1 w/w dimethyl carbonate (DMC): fluorinated ethylene carbonate (FEC) + 0.2 wt.% trimethylboroxine (TMB) electrolyte (Solvionic, France) were used for the HE‐NCM‐ and LCP thin film‐ cathodes, respectively. The electrochemical measurements were performed with the help of a BioLogic (VMP2 Princeton Applied Research) potentiostat. The LCP thin‐film cathodes were charged or discharged in cyclic voltammetry mode in the voltage range of 3.0 – 5.1 V with a 0.1 mV s^−1^ scan rate at room temperature (RT). The HE‐NCM cathode materials were charged or discharged to a defined potential in galvanostatic mode at C/15 rate in the 2.8– 5.1 V range at RT. After charging the cathodes to a high potential (≥ 4.6 V), they were kept under charging for several hours to reach thermodynamic equilibrium, until the current drops typically in the range of 0.1 – 0.5 µA and remains stable. After the electrochemical performance, the cells were disassembled in a glovebox, the cathodes were rinsed in DMC and first dried in a glovebox. For synchrotron measurements at Elettra (Trieste, Italy) and BESSY II (Berlin, Germany), the samples were transferred under UHV conditions by using the UHV suitcases which hold pressure at ≈ 10^−9^ mbar. For synchrotron measurements at Diamond Light Source (Oxford, UK), the HE‐NCM samples were transported under Argon atmosphere followed by their drying under UHV conditions. For the Raman spectroscopic and electrochemical impedance (EIS) measurements, the HE‐NCM materials were pressed on Al_2_O_3_ mesh under the pressure of 3 tons followed by assembling of electrochemical cell (EL‐Cell GmbH, Hamburg, Germany) with a borosilicate glass (Alfa Aesar, U.K) and the Whatman 10 µm filter paper as the separator. Thick lithium foil (0.76 mm) (Alfa Aesar, U.K.) was used as the anode. The BioLogic VSP potentiostat was equipped with an EIS impedance card operating in the frequency range of 1 MHz – 5 Hz, with 6 points in decade and a 50 mV amplitude in a SPEIS‐experiment (staircase potentio‐electrochemical impedance spectroscopy starting at 3.2 V after the 1^st^ full charge–discharge cycle to minimize side‐reactions and, therefore, ensure accuracy in the measurements. All potentials were referenced against Li^+^/Li (‐3.022 V vs SHE).

### XPS, SPES, RPES, and XANES Analysis

The HE‐NCM cathodes, as prepared and after electrochemical charging (discharging) to a defined‐potential, were moved from the glovebox to the UHV systems for photoelectron spectroscopy studies by using various facilities (laboratory and synchrotron sources). All experiments were performed in quasi‐in situ regime, i.e., in vacuo sample transfer without contact to air. XPS measurements were performed in the Darmstadt Integrated System for Fundamental Research (DAISY‐FUN) using a PHOIBOS 150 spectrometer (SPECS Surface Nano Analysis GmbH) and a monochromatic Al Kα (hν = 1486.7 eV) source. The base pressure in the analysis chamber was *p*_xps_ <  × 10^−10^ mbar. The photoelectron spectra were collected at an electron escape angle, θ = 90° with respect to the surface. The binding energies were referred to the Fermi level of Ag (or Au) foil in electrical contact with the sample. The probing depth of XPS is d≈ 3 × λ × sin(θ), where λ(*E*_kin_) was the electron inelastic mean free path, which depends on photoelectron kinetic energy (*E*_kin_) and material properties. The probing depth for XPS using Al Kα radiation lies in the range of 5 − 100 Å. The areas under the spectra and energy positions of the photoelectron peaks were obtained by a weighted least‐squares fitting of model curves of 70% Gaussian and 30% Lorentzian character to the experimentally measured spectra. CasaXPS and XPSPeak41 software packages were used for the fitting procedure. The background was subtracted using a Shirley‐type function. The LCO‐, NCM‐, and LCP‐ thin film cathodes after preparation were transferred under UHV conditions (*p*_base_ <  0^−8^ mbar) for the XPS measurements in DAISY‐BAT. The X‐ray photoelectron spectra of the HE‐NCM powder materials (which do not contain conductive carbon and PVDF) were also measured. In the case of the composite materials, the Ni 2p photoelectron spectra overlap with the F (KLL) Auger peaks contributed from PVDF. This complicates the interpretation of oxidation state of Ni ions when using XPS.

Synchrotron photoemission electron spectroscopy (SPES), soft X‐ray absorption near edge structure spectroscopy (XANES) and resonant photoemission spectroscopy (RPES) experiments were performed at the BACH beamline of CNR at the Elettra synchrotron facility (Trieste, Italy). The treated HE‐NCM composite cathode materials were delivered to the BACH beamline endstation under ultra‐high vacuum (UHV) conditions to avoid exposure to air. The quasi‐in situ Co L_3,2_, Ni L_3,2_, and Mn L_3,2_ XANES were measured in total electron yield (TEY) mode by monitoring the drain current through the sample using a Keithley 428 current amplifier. The energy resolution was better than 250 meV, and the intensities were normalized to the photon flux derived from the total photoelectric current recorded at the last mirror of the beamline. In case of O K‐edge XANES measurements, the incoming beam flux was monitored using a gold mesh placed before the sample. The photon energy scale was calibrated by measuring the kinetic energy of Au 4f photoelectrons from a gold reference sample. RPES data were collected at normal emission using a Scienta R3000 electron energy analyzer. The total energy resolution was set to 300 meV. Binding energies were calibrated to the Au 4f signal from a gold reference in electrical contact with the samples. In RPES spectroscopy, valence band photoemission spectra were collected by varying the photon energy across an absorption edge. This can result in interference between direct photoemission and autoionization channels, leading to a resonant enhancement of the valence band electronic states for the element corresponding to the probed edge.

SPES and soft XANES in partial electron yield (PEY) mode were performed at BESSY II (Berlin, Germany). The PM3 undulator beamline equipped with a soft X‐ray plane‐grating monochromator optimized for elliptical dipole radiation from modern sources enabling the photon energy range of 20 – 2000 eV.^[^ ^58^ ^]^ The SoLiAs integrated UHV system with a SPECS PHOIBOS 150 MCD‐9 electron analyzer was connected to the beamline.^[^ ^59^ ^]^ The binding energies of the spectra had been referenced to the Fermi level of a clean Ag‐polycrystalline foil. Kinetic energies (*E*_kin_) of photoelectrons in the given XANES (PEY) experiments were varied from ≈ 200 to 850 eV enabling non‐destructive depth profiling. The photon energies for the SPES experiments were selected in such a manner to collect the photoelectrons from the same depth.

In addition, Diamond Light Source (Oxford, UK) facilities were used to measure the samples by soft‐ and hard‐ XANES X ray monochromators, enabling to vary photon energies in the range of 100 – 2100 eV and 2.1 – 20 keV. These experiments were performed at the I09 beamline for Surface and Interface Structural Analysis (SISA). The samples were delivered to the beamline in an UHV suitcase filled by Argon. The XANES experiments were performed in TEY mode.

### Raman Spectroscopy

All Raman spectra were acquired on a Jobin‐Yvon Horiba LabRam HRS 800 (600 lines per mm) under the inert argon atmosphere (spectroelectrochemical cell enclosure), using 632.8 nm excitation from a He‐Ne laser and a ×100 objective (Olympus, NA = 0.95). The excitation power was set to 3 mW. The operando spectra were recorded by using a long‐working distance objective (Olympus × 50 LWD, NA 0.55, MPLN).

## Conflict of Interest

The authors declare no conflict of interest.

## Author Contributions

Z.L. thin‐film deposition and XPS, synchrotron measurements at Elettra and Diamond Light Source, review and editing. A.B. – thin‐film deposition, electrochemical characterization of thin‐film batteries, data analysis. M.R. – operando Raman spectroscopy, data analysis. M.M., C.M. synchrotron measurements at BESSY‐II, review and editing. S.M., H.S. chemical synthesis of HE‐NCM, electrochemical measurements. I.P., S.N., E.M., F.B. – synchrotron measurements at Elettra, data analysis, review and editing. S.N., E.M. – calculations of XANES. R.W. – synchrotron measurements at Diamond Light Source, review and editing. R.H. – funding acquisition, review and editing. C.H., L.A., B.M., D.A., W.J. – supervision, funding acquisition, review and editing. C.H., B.M., W.J. discussion on the Raman spectroscopy, electrochemical, and electronic structure aspects, respectively. G.C. – idea, conceptualization and methodology of the research, thin film deposition, planning and performance of the electrochemical, XPS, synchrotron spectroscopy experiments, data analysis, writing of the original draft, review and editing, supervision, funding acquisition.

## Supporting information



Supporting Information

## Data Availability

The data that support the findings of this study are available from the corresponding author upon reasonable request.
