# Supplementary material for: Novel Insights into Enhanced Stability of Li‐Rich Layered and High‐Voltage Olivine Phosphate Cathodes for Advanced Batteries through Surface Modification and Electron Structure Design
Source: Adv Sci (Weinh). 2024 Dec 27;12(7):2413054. doi: 10.1002/advs.202413054 (PMC11831568; doi:10.1002/advs.202413054)
Supplement: Supplementary file 1 — Supporting Information [file ADVS-12-2413054-s001.pdf]

ADVANCED SCIENCE
Open Access

Supporting Information

for *Adv. Sci.*, DOI 10.1002/advs.202413054

Novel Insights into Enhanced Stability of Li-Rich Layered and High-Voltage Olivine Phosphate Cathodes for Advanced Batteries through Surface Modification and Electron Structure Design

*Zhili Liang, Abdulaziz Baubaid, Mariusz Radtke, Maximilian Mellin, Clément Maheu, Sandipan Maiti, Hadar Sclar, Igor Píš, Silvia Nappini, Elena Magnano, Federica Bondino, Robert Winkler, René Hausbrand, Christian Hess, Lambert Alff, Boris Markovsky, Doron Aurbach, Wolfram Jaegermann and Gennady Cherkashinin**

Supporting Information

Novel Insights into Enhanced Stability of Li-Rich Layered and High-Voltage Olivine Phosphate Cathodes for Advanced Batteries through Surface Modification and Electron Structure Design

Zhili Liang,[a] Abdulaziz Baubaid,[a] Mariusz Radtke,[b] Maximilian Mellin,[a] Clément Maheu,[a,⊥] Sandipan Maiti,[c] Hadar Sclar[c], Igor Píš,[d] Silvia Nappini,[d] Elena Magnano,[d,e] Federica Bondino,[d] Robert Winkler,[a] René Hausbrand,[a] Lambert Alff,[a] Christian Hess,[b] Boris Markovsky,[f] Doron Aurbach,[f] Wolfram Jaegermann,[a] and Gennady Cherkashinin.[a,*]

[a] *Institute of Materials Science, Technische Universität Darmstadt, Peter-Grünberg-Str. 2, D-64287, Darmstadt, Germany*
[b] *Department of Chemistry, Eduard-Zintl-Institut für Anorganische und Physikalische Chemie, Technische Universität Darmstadt, Peter-Grünberg-Str. 8, D-64287, Darmstadt, Germany*
[c] *Department of Chemistry, Institute for Nanotechnology and Advanced Materials (BINA), Bar-Ilan University, Ramat-Gan, 5290002, Israel*
[d] *CNR - Istituto Officina dei Materiali (IOM), Strada Statale 14, km. 163,5 in Area Science Park Basovizza, Trieste, 34149, Italy*
[e] *Nanotechnology Research Laboratory, Faculty of Engineering, University of Sydney, Camperdown 2006, Australia*
[f] *Department of Chemistry, BINA - BIU Institute of Nanothechnology and Advanced Materials, INIES - Israel National Institute of Energy Storage (supported by Israel Ministry of Energy and Infrastructures), Bar-Ilan University, Ramat-Gan, 5290002, Israel*

Discussion S1: The impact of SO_2 and NH_3 gases treatment on the properties of HE-NCM cathodes

The reason for choosing NH_3 alone was that upon ammonia treatment, protons are generated from the NH_3, and the surface oxygen is removed to maintain charge balance as Li^+ is partially leached from the surface. We suggest that the result of oxygen elimination from the particle surface is the formation of a spinel-like, oxygen-deficient phase. Overall, NH_3 treatments of Li-rich materials at 400 °C improve discharge capacity of electrodes and decrease the discharge capacity fading and average voltage during cycling in Li-cells. Thermal treatment of HE-NCM with SO_2 gas results in the oxidation of sulfur to S^{6+} valence state, partial reduction of manganese, and the formation of stable nano-sized layer comprising sulfates and sulfites on the surface. The thickness of the protective layer on HE-NCM is several nanometers, as supported by our previous research, where the Li_2SO_4 layer formed on the NCM85 particles due to the SO_2 treatment was several nano-meters.[1S] Our results on the artificial layer formation demonstrated that a too low thickness might not be sufficient to avoid the direct contact between high-voltage cathodes and the electrolytes.[2S] An important conclusion is that the bulk of the material remained unchanged, as confirmed by X-ray and electron diffraction studies. Lithiated $LiMn^{(3+\alpha)}O_{(2-\beta)}$ species formed due to the "non-electrochemical activation" of the Li_2MnO_3 component of integrated HE-NCMs

⊥ *The current address: Nantes Université, CNRS, Institut des Matériaux de Nantes Jean Rouxel, IMN, F-44000 Nantes, France*

(typically, $0.35Li_2MnO_3·0.65LiNi_{0.35}Mn_{0.45}Co_{0.2}O_2$) contribute to the enhanced electrochemical performance of these cathodes.

Discussion S2: Nature of the peak at 533.7 eV in O K XANES of the SO_2 and NH_3 double gas *treated HE-NCM* cathode.

The spectral features in the 533.7 eV energy range are commonly ascribed to Li_2CO_3 and Li_2O,[3S] and LiOH (534 eV,[4S]). In principle, lithium hydroxide could be formed as the result of leaching Li^+ from the surface followed by LiOH conversion to Li_2CO_3 in air.[5S] However, our previous study demonstrated that the amount of LiOH is even smaller in the HE-NCM cathodes treated by NH_3, as compared to the untreated sample.[5S] It is also well-known that LiOH is unstable under X-ray beam in ultra-high vacuum and decomposes into Li_2O and H_2O followed by desorption of water.[6S] In addition, the current experiments exclude contact of the cathodes with air. Our resonant photoemission spectroscopy (RPES) experiments performed by scanning photon energy in the 533 – 535 eV range also could not support LiOH at the surface (Chapter 2.2., Figure 2e). Lithium carbonate is also ruled out, because the C 1s photoelectron spectra of the *untreated*- and *treated- HE-NCM composites* are very similar (Figure 1 h). The absence of Li_2CO_3 and LiOH is also confirmed by Raman spectroscopy (Figure 1l). However, this technique probes the samples deeper, in a few hundreds of nanometres depth. Li_2O is commonly detected by Raman spectroscopy at 520 – 530 cm^{-1}.[7S] Therefore, a slight increase of the Raman signal at ~525 cm^{-1} for the *treated HE-NCM composite* (Figure S5a, Supporting Information) suggest the possibility of Li_2O formation predominately at the surface in accordance with a more surface sensitive XAS. Thus, the comparison of the O K XANES and the Raman spectroscopic results indicates a concentration gradient of the relevant Li_2O species formed upon the HN_3 and SO_2 double gas treatment with a higher content at the surface. Note that Li_2O is widely used in the technology to fabricate LRC with an additional lithium source for a compensation of the irreversible loss of Li during long-term cycling. However, in our work Li_2O was not added upon chemical synthesis of HE-NCM, but Li_2O can purposely formed through NH_3 gas treatment reacting with Li_2MnO_3:[5S]

$$2Li_2MnO_3 + NH_3 + 1.5O\ (lattice) \rightarrow 2LiMnO_2 + Li_2O + 1.5H_2O + NO_{(gas)}\ (at\ 400\ °C)$$

Lithium peroxide (Li_2O_2), which could be formed via another chemical reaction of Li_2MnO_3 with Li_2O at elevated temperatures,[5S] is not revealed by neither O K XANES (typically at 531–532 eV,[3S]) nor Raman spectroscopy (typically O–O peroxide vibration at 780-805 cm^{-1},[7S,8S]).

Discussion S3: Evolution of the O K XANES in the 532 - 533 eV range

A closer examination of the O K-edge XANES of the *treated HE-NCM composite* shows a decrease in the intensity of the shoulder (532 eV – 533 eV) composed from overlapping of the *B* and B^* peaks (Figure 7a(i-ii), Figures S18a(i-iii), S19, Supporting Information). Since the M are oxidized, the peak *B* (~532 eV) associated with the M 3d states should be increased in intensity, thereby manifesting that more holes are formed in the Co 3d (e_g and t_{2g}) and Ni 3d (e_g) states (Figure S1a(iv-vi,xi),(vii-ix), Supporting Information). Therefore, the observed lowering of the shoulder B^* in the 532 eV - 533 eV photon energy range is more probably assigned with decomposition of the N–O bond via $2NO \rightarrow N_{2\,(g)}\uparrow + O_{2\,(g)}\uparrow$,[9S] which may be triggered by a catalytic reaction at the surface modified upon the SO_2 and NH_3 treatment, and which of course depends on the position of the Fermi level. In this regard, one notes that loss of NO_x species after exposure the *treated HE-NCM* cathode to the electrolyte and charging the cathode is also observed in the N 1s photoelectron spectra (Figure S2, Supporting Information). The VB electronic states associated

with the NO molecule and, in particularly, with the O 2p level is expected to be at 2.0 eV - 2.7 eV range and ~9 eV ascribed to the 1π- and $2\pi^*$ - orbitals of N-O, respectively.[10S,11S] However, the photoelectron signal from NO_x is very small, thus, the O 2p orbitals of N-O are probably overlapping with a stronger signal from Li_2O which shows an onset at E_{bin}~4.5 eV in the VB spectra (Figure 2e).

Discussion S4: Electrochemical properties of the treated- and untreated- HE-NCM cathodes

The comparative electrochemical behaviors of HE-NCM cathodes have been thoroughly discussed in our previous work.[12S] Importantly, the *treated HE-NCM* demonstrates a lower hysteresis and lower impedance after the first electrochemical cycle, as compared to the untreated cathode (Figure 6; Figures S11, S12, Supporting Information). Our previous studies on a long time performance of HE-NCMs demonstrated convincingly that the double gas treatment induced more stable cycling at a 1C rate (in full-cells with graphite anodes) in terms of much higher capacity retention of the gas-treated materials (72 % vs. 54 %) after 400th cycle. Moreover, electrodes comprising double-gas treated HE-NCM materials exhibited higher discharge capacities at a 3C-rate, stabilized discharge voltage, lower voltage hysteresis and its evolution with cycling. As expected, impedance of these electrodes is also smaller and grows to a less extent upon cycling than that of untreated ones.[12S]

Discussion S5: Origin of the spectral feature of the O K XANES at 532.7 eV

The empty state at 532.7 eV of the O K XANES is missing in the *untreated HE-NCM* cathode along all charging states, including as-prepared cathode (Figure 7b(i)). It is likely that, the B^* shoulder at 532.7 eV of O K XANES of the *treated HE-NCM* cathode is associated with the decomposition of NH_3 and formation of NO_x. Note that the energy the B* shoulder coincides with an empty state ascribed to the N–O bond (Figure 7a(i,ii)). Although the N-O bond vanishes at 4.5 V charging potential (Figure 7a(iii)), in agreement with a trend in the N 1s photoelectron emission (Figure S2(iv), Supporting Information), the relevant state occurs again by charging *treated HE-NCM* to 4.6 V vs. Li^+/Li. This observation might mean reversible catalytic process at the electrolyte-cathode interface, potentially involving nitrogen and oxygen dissolved in the electrolyte, although the inverse chemical reaction, namely: $N_{2(g)} + O_{2(g)} \rightarrow 2NO$ is unfavourable due to the high temperatures needed for the synthesis.

Discussion S6: Oxidation state of Ni, Co and Mn ions at 4.8 V

The expected highest oxidation state of Ni, Co and Mn in an conventional $Li_{1.0}[Ni^{2+}, Mn^{4+}, Co^{3+}]O_2$ (NCM) cathode material is 4+. Mn is assumed to be electrochemically inactive, whereas Ni and Co are consecutively oxidized upon charging the cathode from N^{2+} via Ni^{3+} to Ni^{4+} and from Co^{3+} to Co^{4+}. However, the revealed oxidation of HE-NCM is lower than 4+ in both the *treated*- and *untreated- HE-NCM* cathodes. This finding is supported by the Ni L-edge XANES, where the *B/A* < 1 intensity ratio indicates an oxidation state lower than 4+ (Ni L XANES of tetravalent Ni is shown in Figure S22, Supporting Information), in agreement with the relative shifts of the Co K- and Ni K- XANES, which are smaller (Δ_{CoK} ~2.4 eV and ΔE_{NiK} ~3.2 eV) than expected for 3+ and 4+ oxidation states, respectively (Figures S15, S16, Supporting Information). As an example, $Li_xNi_{1/3}Co_{1/3}Mn_{1/3}O_2$ delithiated to x=0.33,[13S] corresponding to ~4 V charging state,[14S] exhibits a similar value of Δ_{CoK} ~2.4 eV, whereas a maximal chemical shift of about 4 eV was detected in Ni K XANES for various Ni oxides with the Ni^{2+} and Ni^{4+} valence states.[15S] Oxidation state of Mn: between 3.5+ and 4+ for the *untreated HE-NCM* (Figure 5) and between 2+ and 3+ for the *treated HE-NCM* (Table II).

Discussion S7: Electronic structure of $LiCoPO_4$ (LCP) olivine-type structure cathode material near Fermi level

The occupied states of LCP near the E_F (Figure 9a) are characterized by four spectral features associated with the t_{2g} and e_g states of Co (A feature), the Co 3d hybridized with O 2p bonding and antibonding states (B feature), and the strongly bound states of oxygen and phosphor (C and D peaks). The VB spectral features are sharp and well separated from each other, which is a sign of a lower M 3d – O 2p hybridization as compared to HE-NCM (Figure S9a(i),b(i), Supporting Information). O K-edge XANES of as-prepared LCP thin film shows the most intense peak situated at ~5 eV from the E_F, occurred due to the electron transition from the O 1s core-level to the empty O 2p band. The broadness of the O K- edge of phosphates, as compared to layered structure oxides, reflects a strong covalent oxygen–phosphor bonding in the PO_4^{3-} unit.[2S]

Discussion S8: The general remarks on design of artificial interface

- The cathode and the protective layer should be chemically compatible to avoid the formation of the interface which might be not Li^+ ion conducting. Preliminary inputs on chemical compatibility between the materials, which are in contact with each other, can be estimated by considering the electrochemical potential and ionization potential of materials before their contact;

- the top of the density of occupied electronic states of the protective layer, which is the valence band (VB) maximum, should be situated sufficiently below of E_F of the cathode at a highest voltage cut-off envisaged for battery cycling. Such a condition will allow to avoid undesirable electronic charge transfer between the cathode and protective layer during cycling that would lead to the formation of cathode-electrolyte interface, which might be not Li^+ ion conducting.

Discussion S9: The general remarks regarding separation of delithiation/lithiation reactions from interfacial chemical reactions for various cathode materials by using electron spectroscopy

It is assumed that the cathodes are sufficiently electronic conductive. In this case it would be possible to

- determine quantitatively the energy distribution of the electronic states of the initial cathode with respect to E_F, as well as the work function (WF) and ionization potential (IP);
- measure the valence band maximum (VBM), WF, IP at a certain charging/discharging potential.
- determine the value of the VBM shift towards E_F upon delithiation of the cathode, is a sign of removal of valence electron from the lattice, i.e. oxidation process of cations or anions that can be confirmed by XPS, XAS, etc.;
- determine the E_F shift in the opposite direction upon delithiation of the cathode, is a sign of reduction of cations or anions, which might be related to side-reactions at the interface that can be confirmed by XPS, XAS, etc.;
- compare the measured WF, IP with oxidation potentials of the electrolytes components. The vicinity of the WF, IP with the electronic levels of the electrolyte is a sign of the interfacial charge transfer between the cathode and the electrolyte;
- use depth-resolved photoemission experiments (angle-dependent, photon energy variation) in addition to separate delithiation/lithiation processes from interfacial chemical/ catalytic reactions.

Note, that although it is near impossible to determine the absolute value of the WF due to its high sensitivity to the surface conditions, such as lateral inhomogeneity, dipole layer formation, etc., our investigations demonstrated a systematic increase (decrease) of the WF upon consecutive delithiation (lithiation) of $LiCoO_2$ and $LiCoPO_4$ thin-film cathode materials,[2S,16S] as well as of the Li-rich NCM composite materials studied in the current work. Further, the valence electronic states are sufficient broad in the energy, usually a few eVs (Fig. 2, and Figs. S6, S8, S9, Supporting Information), whereas the change of the electrochemical potential upon delithiation/lithiation is in the range of less than 2 eV (Table I).[2S,16S] This enables us to determine with high enough precision the pinning/crossing of a specific occupied electronic state by the electrochemical potential upon delithiation of the cathodes.

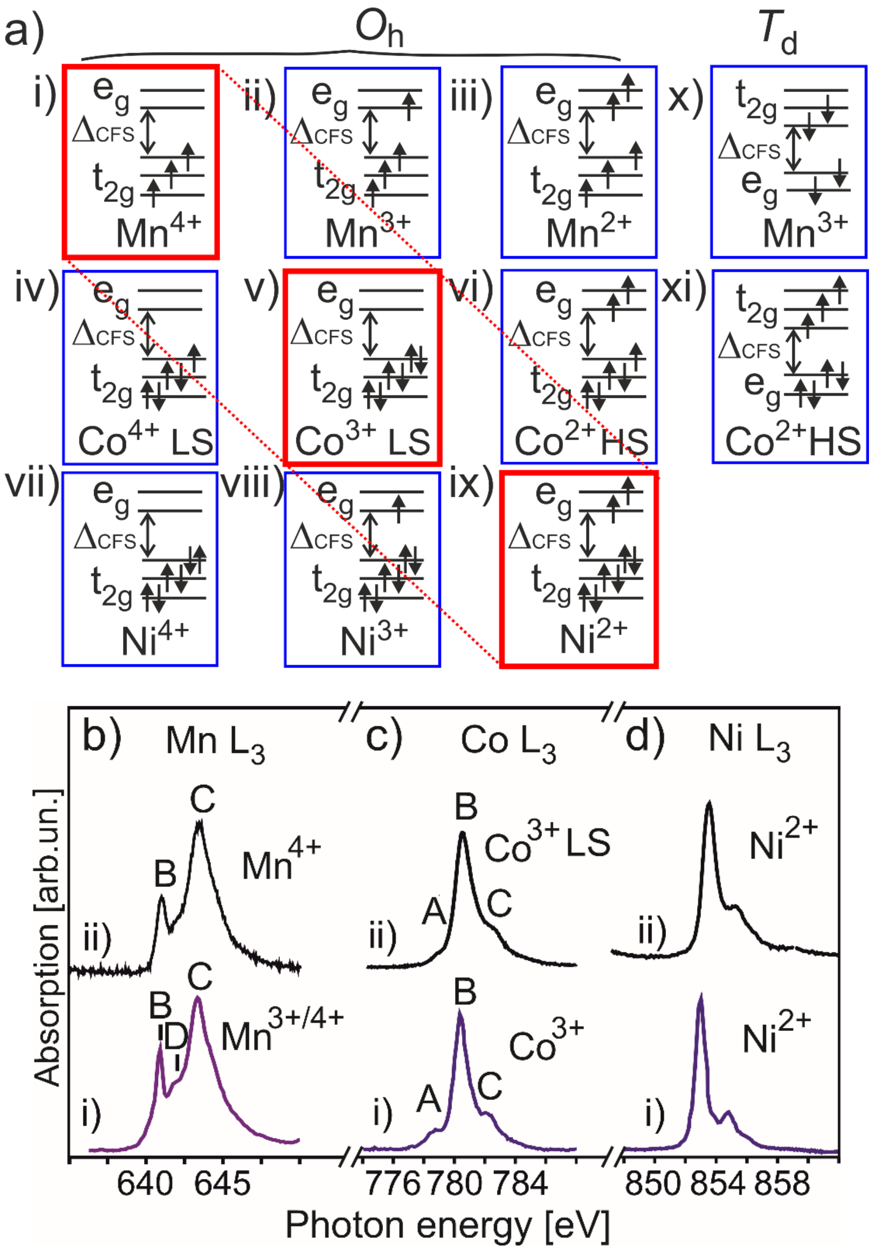

Figure S1. a) The possible ground state electronic configurations of the M 3d states in octahedral (*O*$_h$) and tetrahedral (*T*$_d$) symmetries with O 2p level; Δ_{CFS} is crystal field splitting equals to the energy difference between t_{2g} and e_g levels. The electronic configurations of as-prepared *untreated HE-NCM* are shown in the red frames. (b-d) Mn L_3 (b), Co L_3 (c), and Ni L_3 (d) XANES of the *untreated HE-NCM composite* (i) and $LiNi_{0.2}Co_{0.7}Mn_{0.1}O_2$ thin-film cathode (ii).

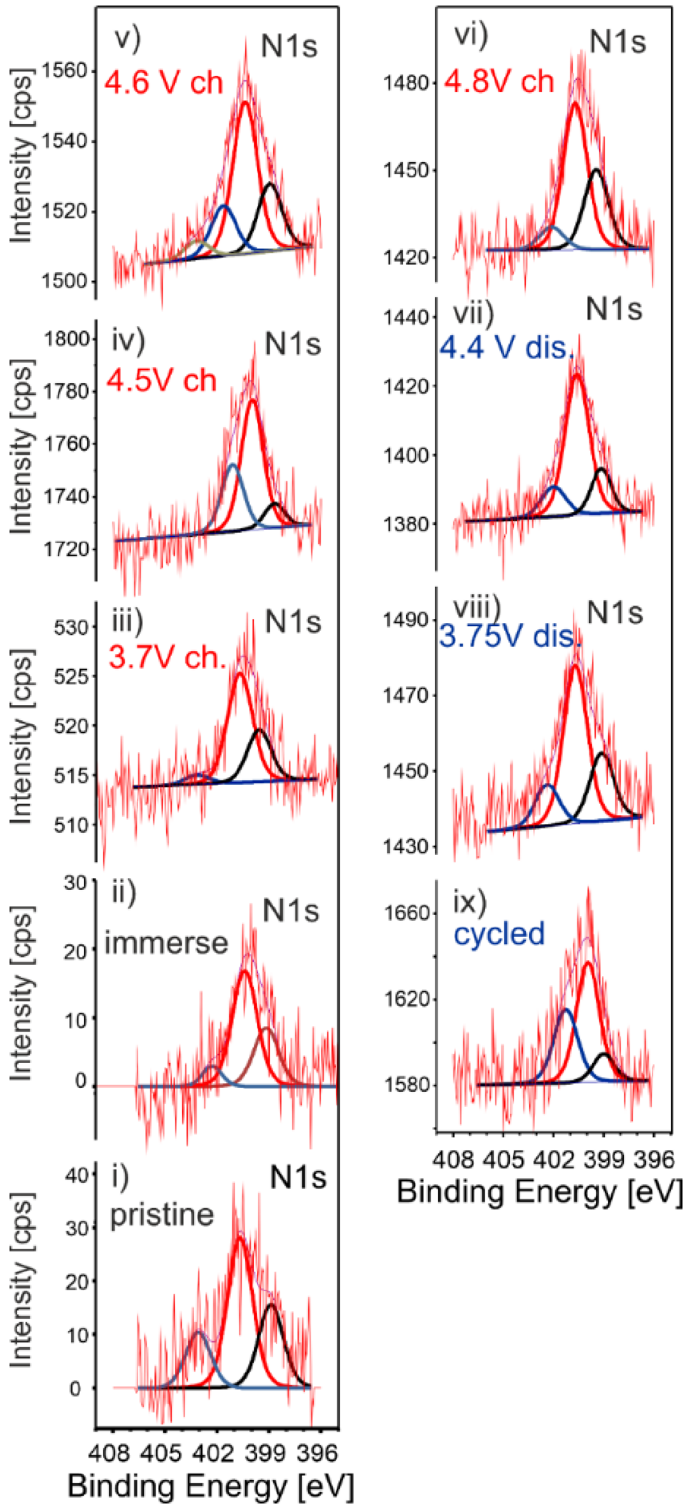

Figure S2. N 1s photoelectron spectra of the *treated HE-NCM* cathode versus (dis-)charging potential. The pristine (i), after soaking into electrolyte (ii), charging states of 3.7 V (iii), 4.5 V (iv), 4.6 V (v), 4.8 V (vi), discharged states of 4.4 V (vii) and 3.75 V (viii), after the first electrochemical cycle (ix). The spectral features at ~399 eV, 400.7 eV and ~403 eV are ascribed to NH_x, NH_3 and N–O, respectively.

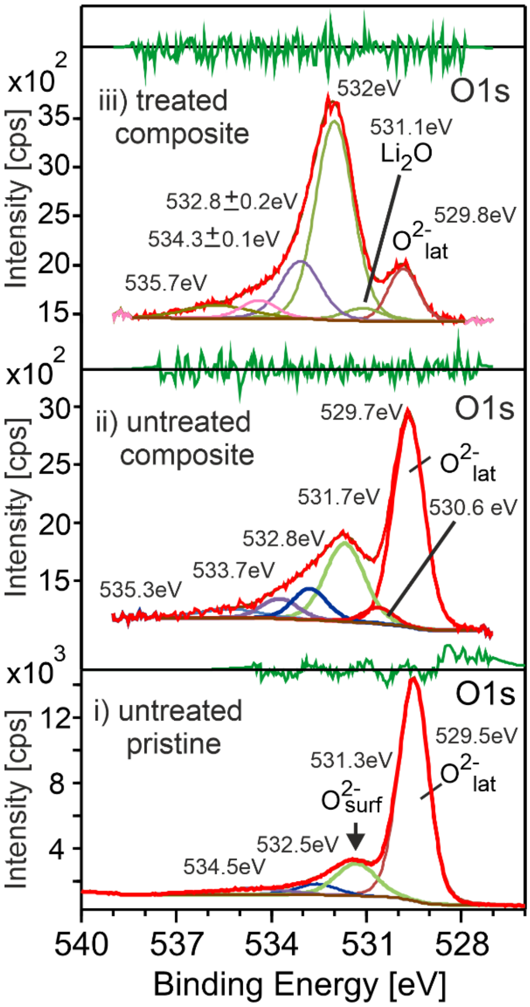

Figure S3. The O 1s photoelectron spectra of *untreated HE-NCM* (i), *untreated HE-NCM composite* (ii), *treated HE-NCM composite* (iii). The photoemission at 531.1 eV of the *treated HE-NCM composite* (iii) is ascribed to Li_2O in accordance with binding energy of Li_2O in the Li 1s spectrum (see Ref.[17S]) and the difference between the O 1s and Li 1s photoemissions: Δ(O1s – Li1s)=474.8 eV.[18S]

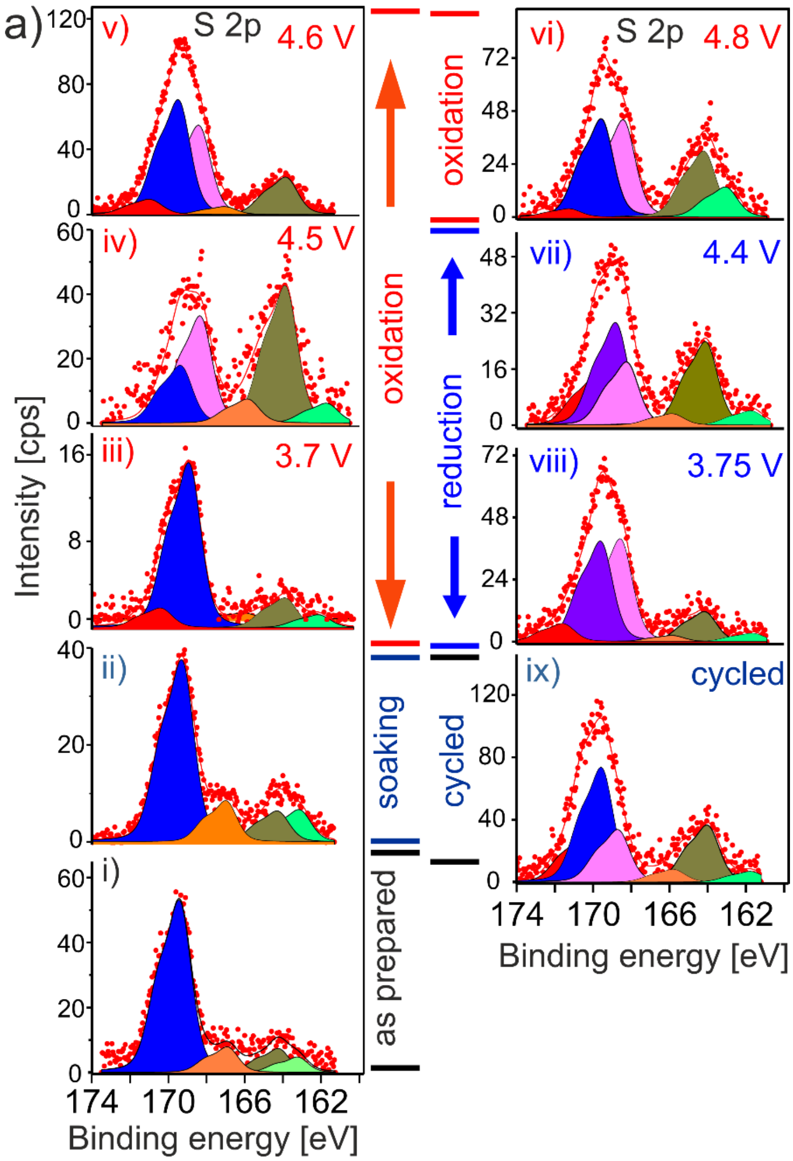

Figure S4. S 2p photoelectron spectra of the *treated HE-NCM* cathode versus charging and discharging potentials. As-prepared sample (i), after soaking into electrolyte (ii), the charged states: 3.7 V (iii), 4.5 V (iv), 4.6 V (v), 4.8 V (vi), discharged states: 4.4 V (vii) and 3.75 V (viii), after the first electrochemical cycle (ix). A strong contribution at 169.4 eV is ascribed to SO_4^{-2} bond. The features at ~167 eV, ~164 and ~162 eV are ascribed to SO_3^{-2}, S_x and Li_2S, respectively. The photoemission at ~168 eV is probably assigned with a metal (M)-SO_4 bond due to impurities and other sulphur-oxygen related moieties.[17S]

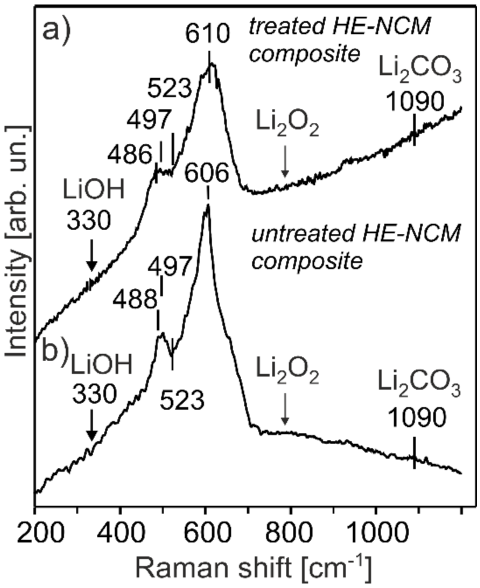

Figure S5. Raman spectra (hv=632 nm) of the as-prepared *treated*- (a) and *untreated*- (b) *HE-NCM* cathodes. No contribution from Li_2O is observed for the *untreated HE-NCM* (b). An intensity increase in the 515 – 525 cm^{-1} range (a) could be attributed to the presence of Li_2O in the *treated HE-NCM composite*. The vertical lines indicate frequency regions of different MO_x vibrations associated with Li_2MnO_3 (~488 cm^{-1}), $LiMO_2$ (491 – 497 cm^{-1}; 518 - 614 cm^{-1},[19S]), LiOH (330 cm^{-1},[20S]) and Li_2CO_3 (1090 cm^{-1}).

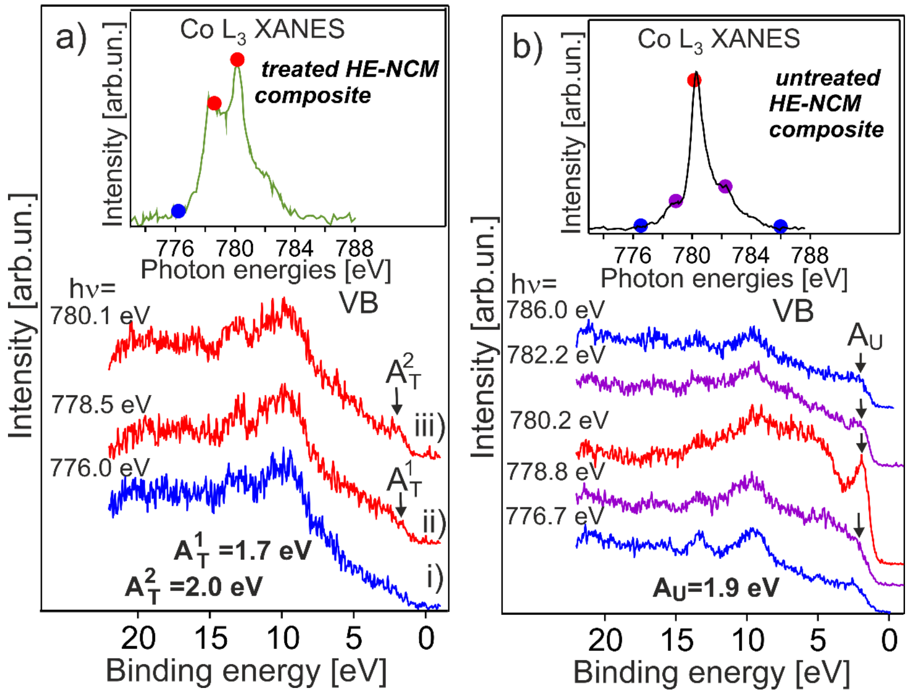

Figure S6. RPES valence band (VB) photoemission spectra of the as-prepared *treated*- (a) and *untreated*- (b) *HE-NCM* cathodes measured by scanning photon energies in the 776 eV – 786 eV range. The solid circles in the insert show photon energies used for the VB measurements. Photon energies (eV): a) (i) 776.0, (ii) 778.5, (iii) 780.1; b) (i) 776.7, (ii) 778.8, (iii) 780.2, (iv) 782.2, (v) 786.0. The VB spectra collected at the Co L_3-edge of the highest intensity show the pronounced resonance (shown in red). a) the Co 3d resonance at A^1_T =1.7 eV and A^2_T =2.0 eV for the *treated* cathode is mostly associated with the Co^{2+} and Co^{3+} oxidation states, respectively. b) A_U=1.9 eV is the Co 3d resonance for Co^{3+} state in the *untreated* cathode.

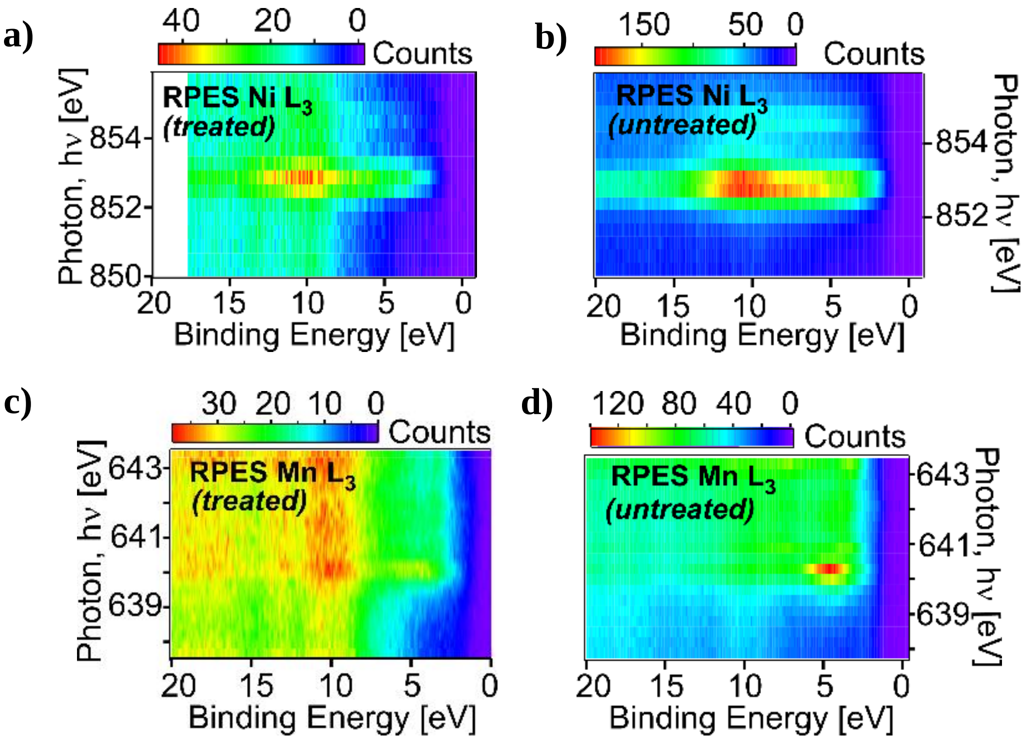

Figure S7. The 2D images of photon energies versus binding energies of the as prepared *treated*- (a, c) and *untreated*- (b, d) *HE-NCM* cathodes. The VB spectra are collected in photon energies range of 849 eV – 856 eV (Ni L_3 XANES) (a, b) and 637 eV – 646 eV (Mn L_3 XANES) (c, d).

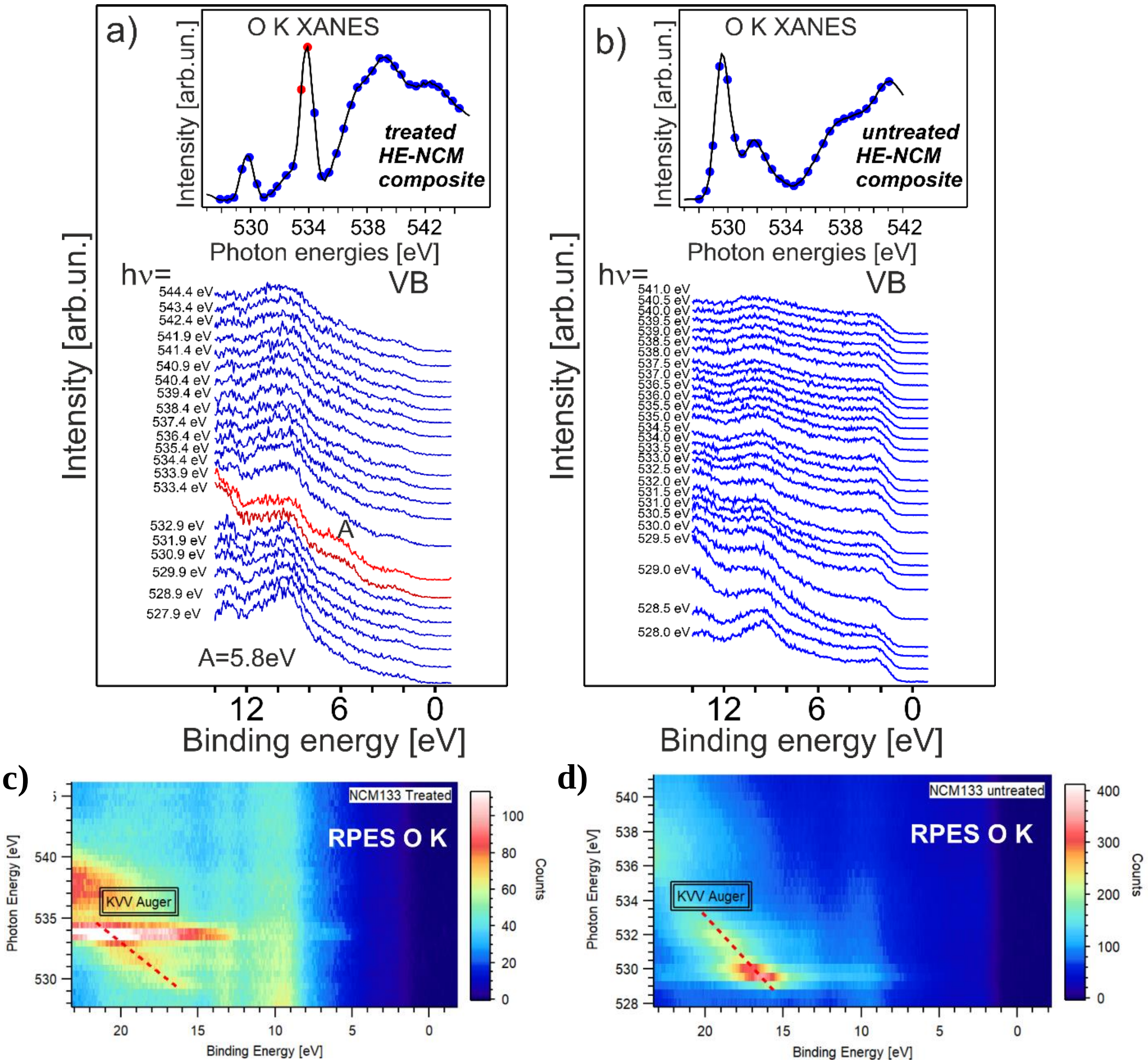

Figure S8. Valence band (VB) photoemission spectra of the as-prepared *treated-* (a) and *untreated-* (b) *HE-NCM* cathodes collected in the 528 eV – 544.5 eV photon energy range. The solid circles in the insert show excitation energies. The A peak at the binding energy (BE) of ~ 5.8 eV (a) is mostly assigned with an empty state of the non-bonding O 2p orbital. The 2D images of photon energies vs BE of the valence band spectra measured in the 0 eV – 24 eV BE range. The O (KVV) Auger transition above binding energy of 15 eV (c, d) is labelled by the dashed line.

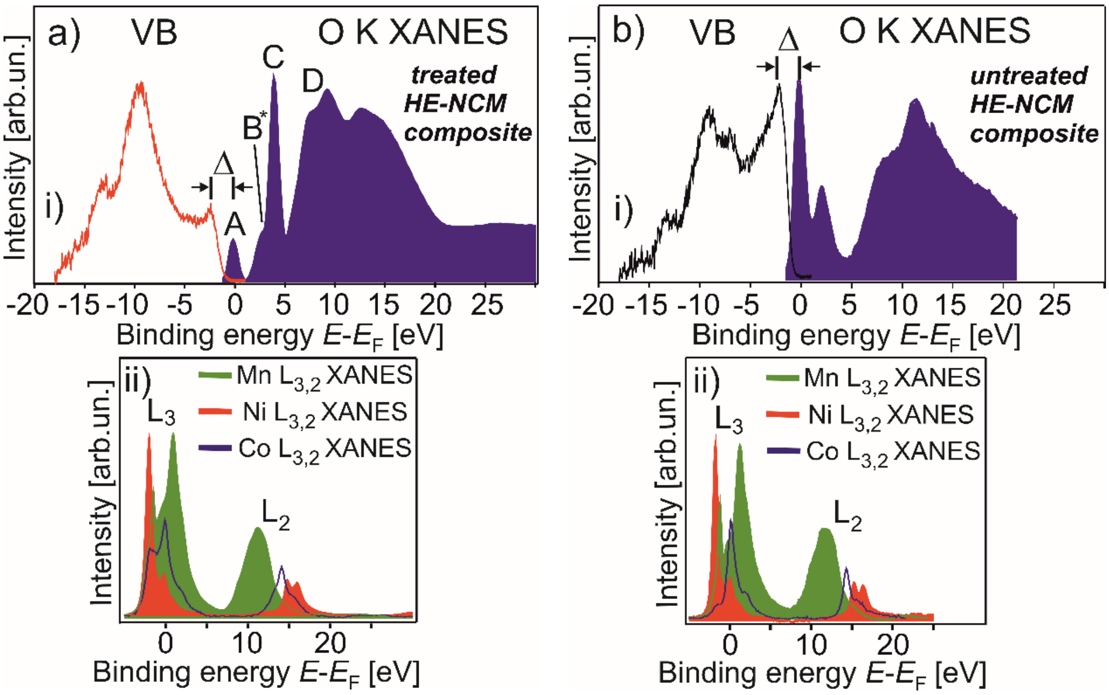

Figure S9. The electronic structure of the as prepared *treated*- (a) and *untreated*- (b) *HE-NCM* cathodes near the Fermi level (E_F=0) obtained from photoemission experiments. (a, b) Off-resonance valence band (VB) photoemission (hv=1486.7 eV) and O K XANES (i). The valence band maximum (VBM) is determined by a linear extrapolation of the onset of the valence band emission to the baseline. The valence band maxima (VBM) are 1.1 $\pm$ 0.1 eV and 0.85 $\pm$ 0.1 eV for the *treated*- and *untreated*- cathodes, respectively. Ni $L_{3,2}$, Co $L_{3,2}$, Mn $L_{3,2}$ XANES plotted vs. the unified energy scale (ii). Δ ~2.5 eV [a(i),b(i)] is the difference between the occupied states and unoccupied M 3d – O 2p hybridization states.

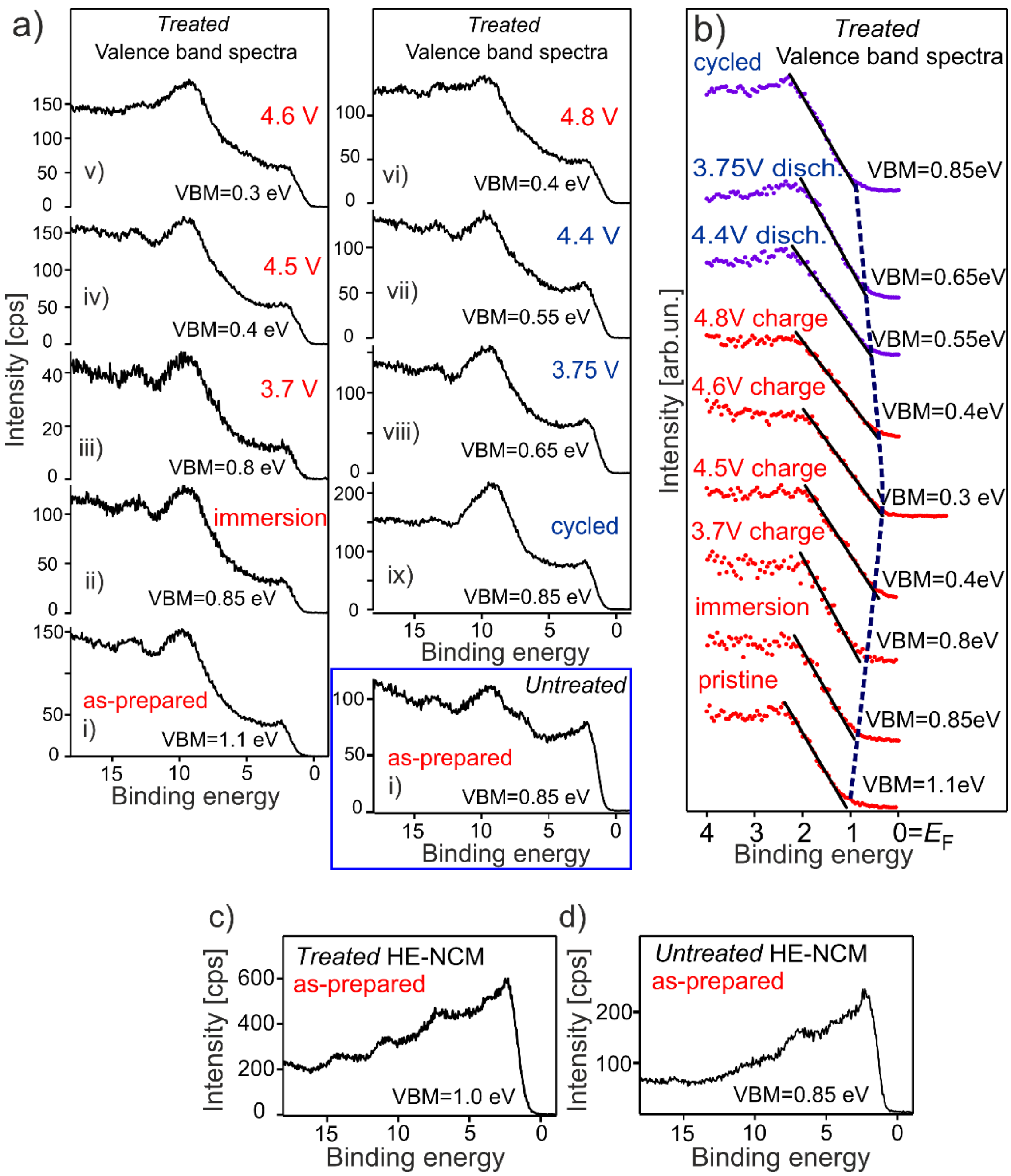

Figure S10. (a, b) The evolution of the valence band (VB) photoelectron spectra (Al Kα, hv=1486.7 eV) of the t*reated HE-NCM* cathode material versus charging/discharging potential. a) The pristine (i), after soaking into electrolyte (ii), charging states of 3.7 V (iii), 4.5 V (iv), 4.6 V (v), 4.8 V (vi), discharged states of 4.4 V (vii) and 3.75 V (viii) and after the first electrochemical cycle (ix). The Fermi level (E_F) is at zero. The valence band maximum (VBM) is determined by the linear extrapolation of the onset of the valence band emission to the baseline. The VB spectrum of as-prepared *untreated HE-NCM* cathode is shown in the insert (the blue frame) (a). b) VBM is decreased upon delithiation and increased again upon lithiation of the cathode, thereby evidencing a shift of E_F towards the occupied electronic states with delithiation of the HE-NCM cathodes and the revers behaviour upon lithiation of the cathodes. c,d) VB photoelectron spectra of the *treated HE-NCM* (c) and *untreated HE-NCM* (d) samples which do not contain conducting carbon and binder.

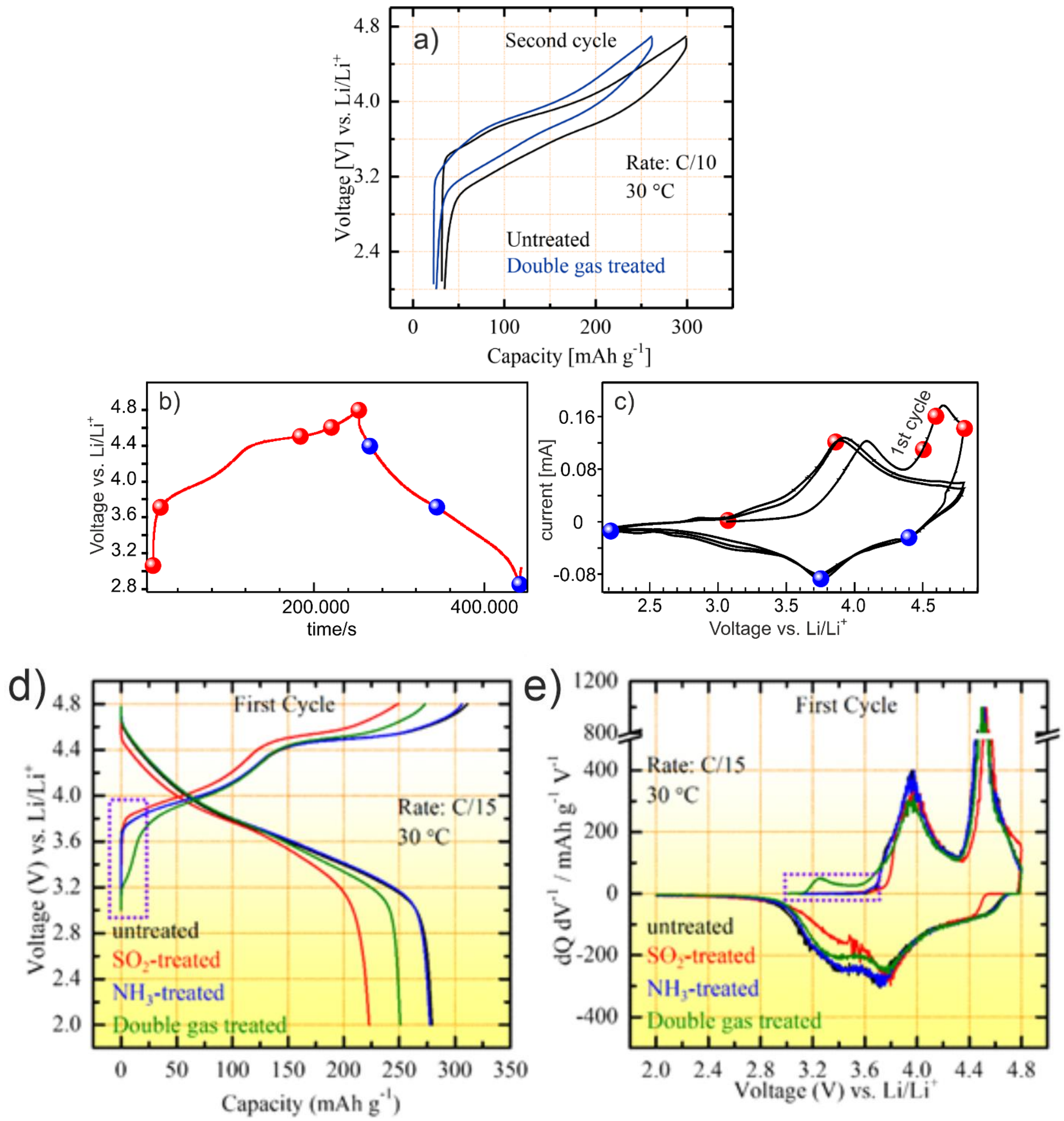

Figure S11. Electrochemical characteristics of HE-NCM double-gas (SO_2 and NH_3) treated composite in 1M $LiPF_6$ in EC-EMC solutions: a) the voltage profile of the 2nd charge-discharge cycle; b) the voltage profile vs. time measured in galvanostatic mode for the 1st cycle; c) cyclic voltammetry. The charging and discharging potentials for XPS, SPES, soft- and hard- XANES are labelled by solid red (charging) and solid blue (discharging) circles. d, e) the voltage profiles (d) and dQ/dV vs. voltage plots (e) of the first charge-discharge cycles measured from electrodes comprising the untreated, SO_2 treated, NH_3 treated, and double-gas treated HE-NCM cathodes (adopted from Ref. 12S). The discharge specific capacity of 250 mAh g^{-1} for SO_2 and NH_3 double gas treated electrodes is achieved at a C/15 rate by using coin-type cells. The capacity retention is 70% for the double gas treated cathode vs. 29% for the untreated one.

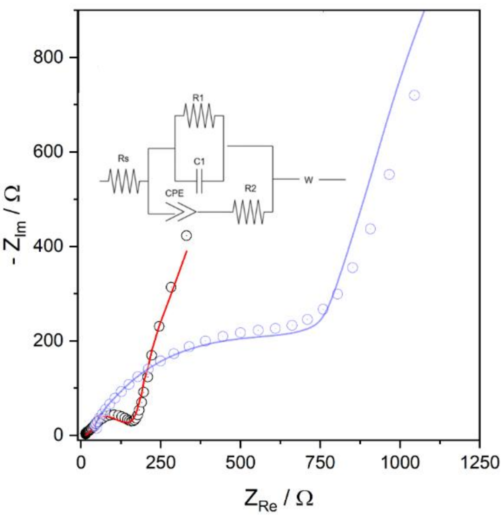

Figure S12. Electrochemical impedance spectra of the *untreated*- (open blue) and *treated*- (open red) *HE-NCM composites* after one electrochemical cycle shown in the Nyquist plot. The insert shows equivalent-circuit comprised of Rs (electrolyte resistance), R1 (electrode surface resistance), R2 (electrode bulk resistance), CPE (constant phase element) due to Li^+ ion loss upon discharge, and C1 (ion trapping due to the intercalation), as well as W (Warburg impedance) responsible for ion mobility. The fitted results are plotted in lines. Semi-circles at high-frequency region corresponds to the migration of Li^+-ion through the electrode thin films,[21S] and they are represented by a constant-phase-element due to the anisotropic character of this movement. The depression of semi-circles is additionally caused by the charge-transfer process due to trapping the Li^+-ions within the bulk of the electrode material.[22S]

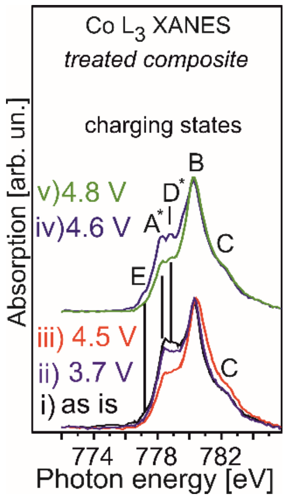

Figure S13. Evolution of Co L_3 XANES of the *treated*- (a) and *untreated*- (b) *HE-NCM* cathodes versus a charging state. As-prepared are labelled by “as is” (i). A steady decrease in the intensity of the low energy A^* shoulder of the Co L_3 XANES associated with the Co^{2+} state (a) evidences oxidation of the divalent Co in the *treated* cathode. The E and D^* intensities inherent to Co^{2+} state decreased [a(iii)] and increased [a(iv)] upon charging the treated cathode to 4.5 V and 4.6 V, respectively (see the Co L_3 XANES calculations in Figure 1f).

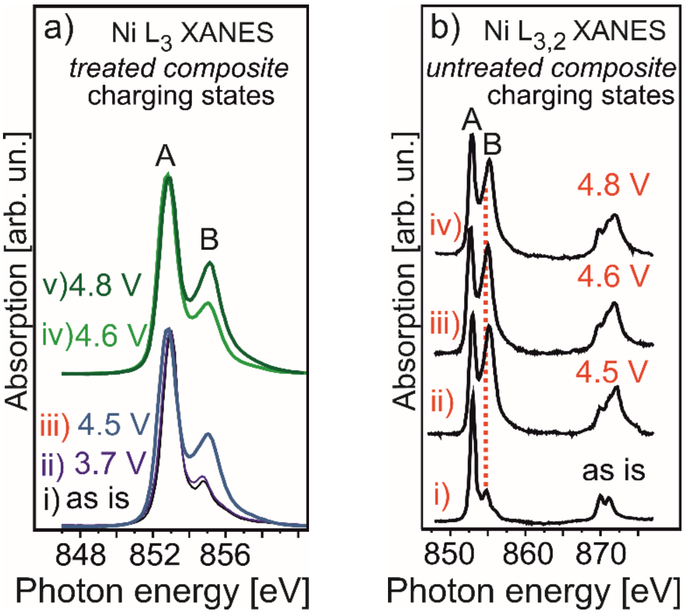

Figure S14. The evolution of Ni L XANES of the *treated*- (a) and *untreated*- (b) *HE-NCM* cathodes versus a charging state. As-prepared samples are labelled by “as is” (i). The B peak position shift upon charging the cathodes: 854.7 eV [a(i)] and 854.8 eV [b(i)]); 854.7 eV [a(ii)]; 855.0 eV [a(iii)] and 855.2 eV [b(ii)]; 855.0 eV [a(iv), b(iii)]; 855.1 eV [a(v)] and 855.2 eV [b(iv)]. The intensity increase of the B shoulder and its shift to higher energies are due to an increase in the oxidation state of Ni. Upon discharging, the B decreased in the intensity and shifted in the opposite direction, see Figure 4b: 855.0 eV [c(vi)]; 854.6 eV [c(vii)]; 854.7 eV [c(viii)], thereby confirming reduction to the Ni^{2+} state in the *treated* cathode.

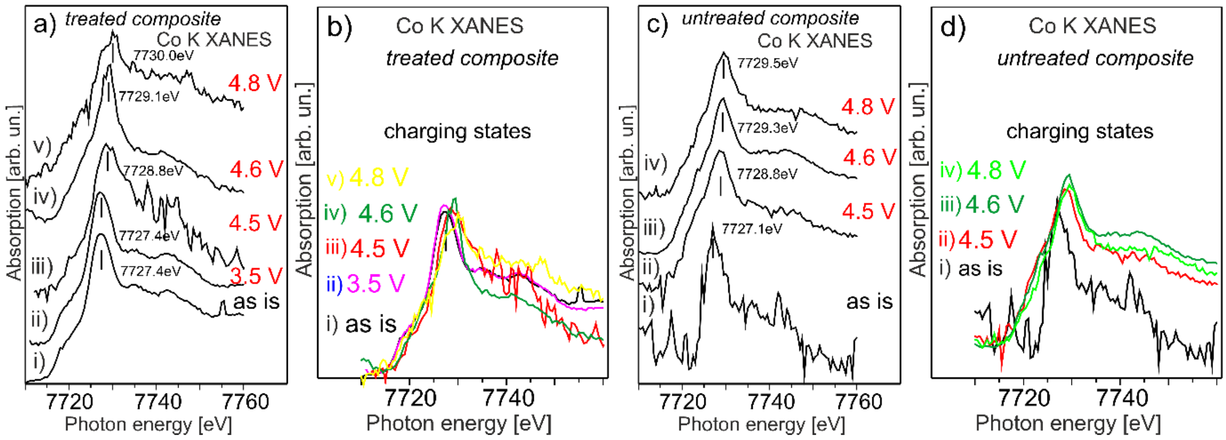

Figure S15. The evolution of Co K XANES of the *treated*- (a, b) and *untreated*- (c, d) *HE-NCM* cathodes versus a charging state. As prepared (i), charged to: 3.5 V [a(ii), b(ii)], 4.5 V [a(iii), b(iii), c(ii), d(ii)], 4.6 V [a(iv), b(iv), c(iii), d(iii)] and 4.8 V [a(v), b(v), c(iv), d(iv)]. The shift of Co K XANES to higher energies supports oxidation of Co.

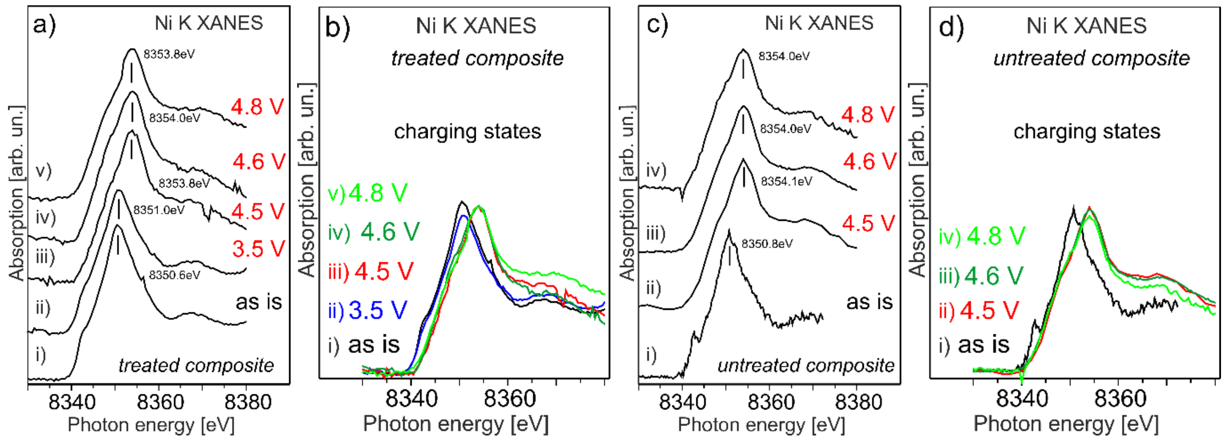

Figure S16. The evolution of Ni K XANES of the *treated*- (a, b) and *untreated*- (c, d) *HE-NCM* cathodes versus a charging state. As prepared (i), charged to: 3.5 V [a(ii), b(ii)], 4.5 V [a(iii), b(iii), c(ii), d(ii), e(iii)], 4.6 V [a(iv), b(iv), c(iii), d(iii)] and 4.8 V [a(v), b(v), c(iv), d(iv)]. The shift of Ni K XANES to higher energies supports oxidation of Ni.

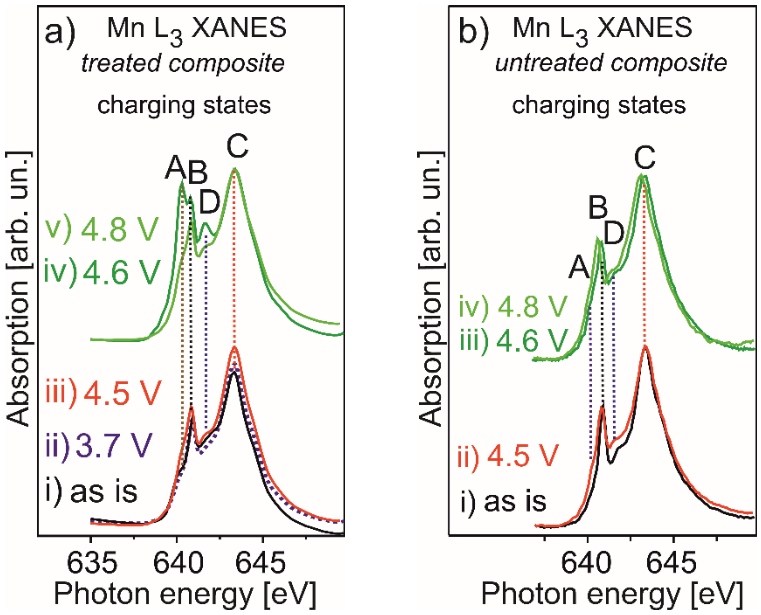

Figure S17. The evolution of Mn L_3 XANES of the *treated*- (a) and *untreated*- (b) *HE-NCM* cathodes versus a charging state. As-prepared are labelled by “as is” (i). The increase in intensity of the A (~640.2 eV), B (640.8 eV) and D (641.8 eV) shoulders (a) is due to the contribution of Mn^{2+} and Mn^{3+} (see theoretical calculations in Figure 1e).

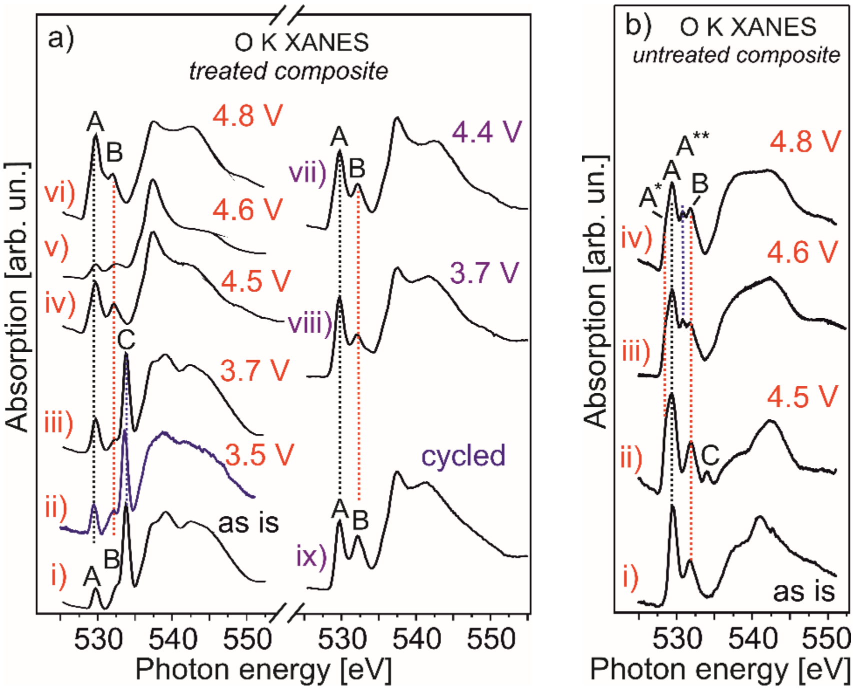

Figure S18. The evolution of O K XANES of the *treated*- (a) and *untreated*- (b) *HE-NCM* cathodes versus charging [a(i-vi), b(i-iv)] and discharging [a(vii-viii)] during the first electrochemical cycle with the upper cut-off of 4.8 V. As-prepared [a(i),b(i)], charged to: 3.5 V [a(ii)], 3.7 V [a(iii)], 4.5 V [a(iv),b(ii)], 4.6 V [a(v),b(iii)], 4.8 V [a(vi),b(iv)]; discharged to: 4.4 V [a(vii)], 3.7 V [a(viii)], cycled [a(ix)]. The C peak intensity is not markedly changed by charging the *treated* cathode to 3.5 V and 3.7 V.

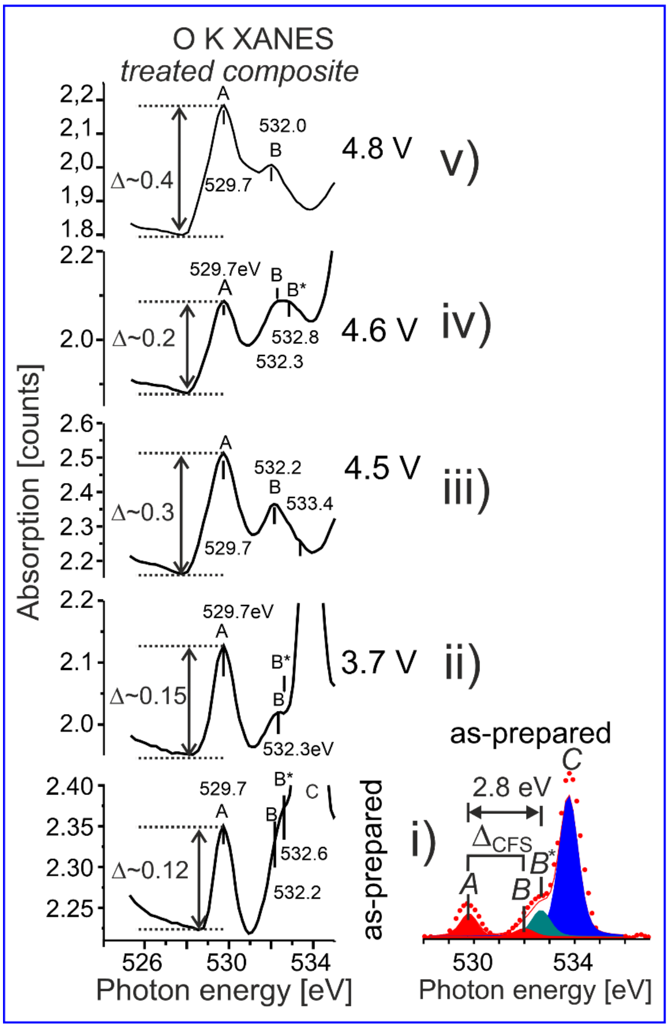

Figure S19. O K XANES of the *treated HE-NCM* cathode versus the charging state. The fitted O K XANES of as-prepared cathode is shown on the right side. The A and B peaks are associated with M 3d (t_{2g}) and (e_g) states, respectively, hybridized with O 2p state (lattice oxygen). Δ_{CFS} is crystal field splitting (see Figure 1a for details). The B^* feature, overlapped with the B and C peaks, is more probably assigned with a small amount NO_x. The C peaks is contributed mostly from Li_2O. An increase of the A and B peaks intensity evidences that more holes are formed in the 3d state, whereas a decrease of the A and B intensity is mostly due to a reduction of the oxidation state of M 3d.

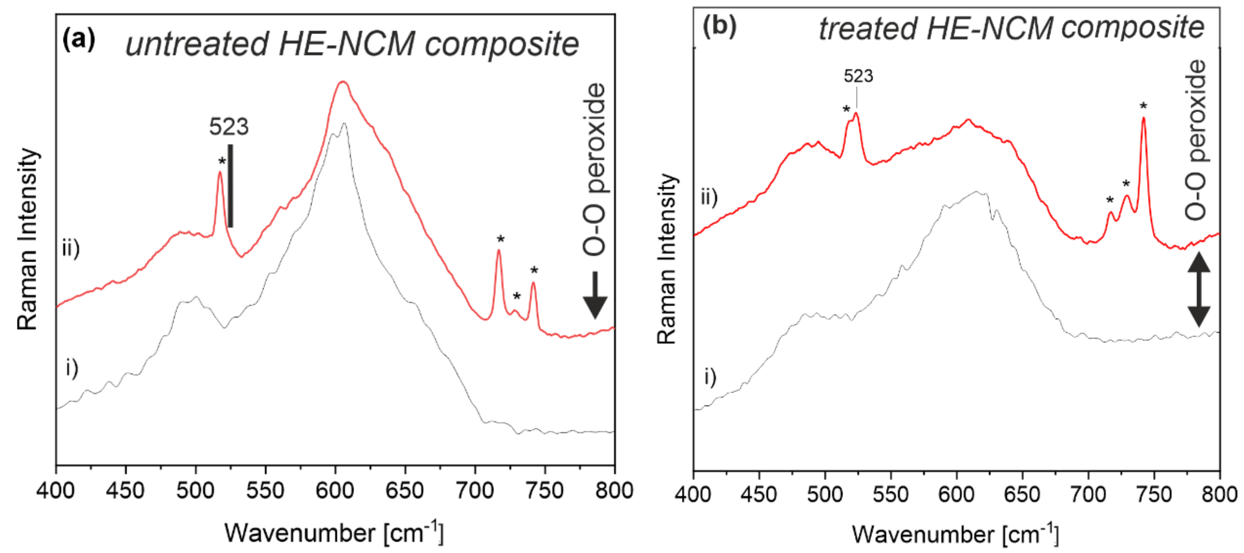

Figure S20. Raman spectra at 632 nm excitation of the *untreated*- (a) and *treated*- (b) *HE-NCM* cathodes. Spectra were recorded for the pristine state (i) and under operando conditions at the 4.5 V charging state (ii). No contribution from Li_2O is observed for the pristine *untreated HE-NCM* (a,i). A slight vanishing of the valley in the 515 – 525 cm^{-1} range of the *treated HE-NCM composite* (b,i) might be assigned with Li_2O. At the charging state (a,ii, b,ii), a minor contribution of Li_2O in the *untreated HE-NCM composite* is evidenced as shoulder at 523 cm^{-1} (Ref.[7S]) of the electrolyte species (517 cm^{-1}) (a,ii). In the treated cathode, Li_2O feature at 523 cm^{-1} is more pronounced (b,ii). Li_2O_2 (peroxide) is not detected (its frequency range is shown by the arrow). Asterisks (*) label electrolyte-related features.

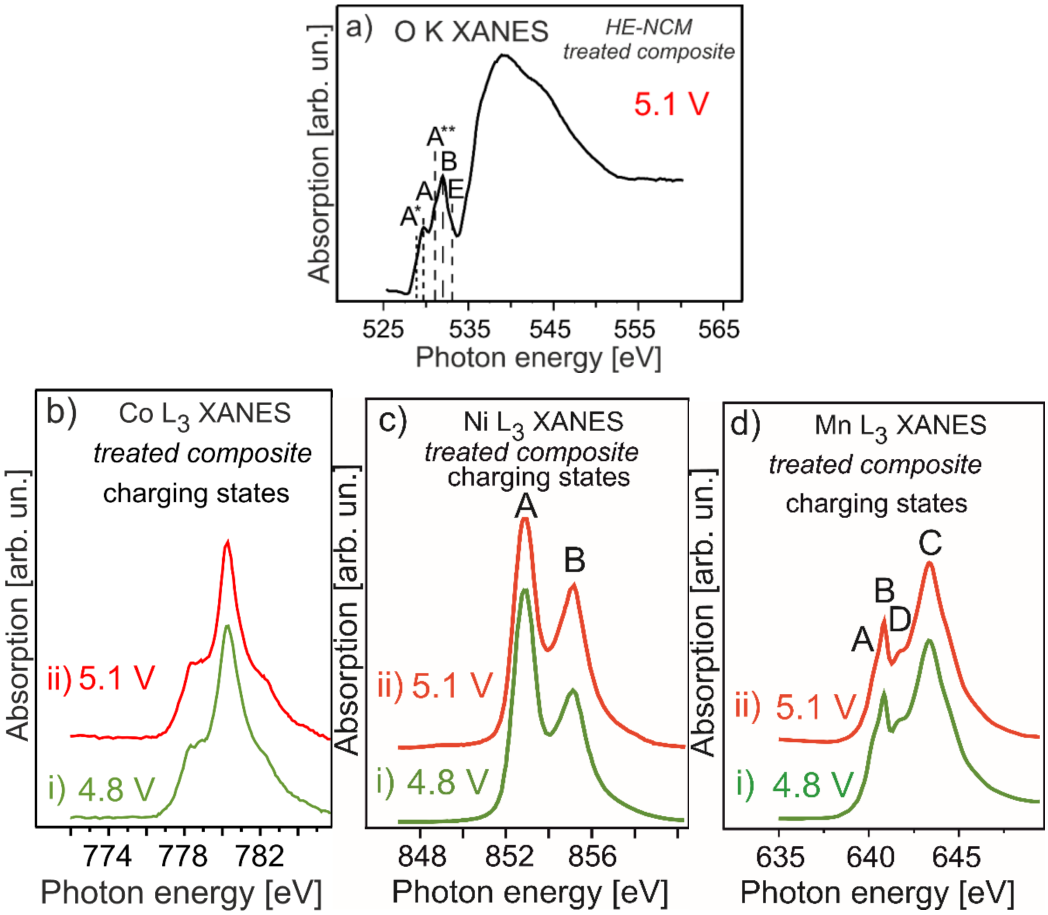

Figure S21. a) O K XANES of the *treated HE-NCM* cathode charged to 5.1 V versus Li^+/Li. A^*=528.8 eV, A=529.7 eV, A^{**} =531.1 eV, B=532.0 eV, E=533.1 eV are ascribed to the O^- hole formation, M 3d (t_{2g}) - O 2p hybridized states, O_2 species overlapped with contribution from C=O groups, M 3d (e_g) - O 2p hybridized states overlapped with contribution from O–C=O groups, carboxylic group overlapped with contribution from Li_2CO_2, respectively. b-d) Co L_3 (b) Ni L_3 (c), and Mn L_3 (d) XANES of the *treated HE-NCM* cathode charged to 4.8 V (i) and 5.1 V (ii). b) No visible changes are observed in Co L_3 XANES vs. the charging states. c) An increase of the B shoulder in Ni L_3 XANES at 5.1 V indicates a higher oxidation state of Ni, as compared to 4.8 V. d) A slight intensity decrease of the D feature in Mn L_3 XANES of the cathode charged to 5.1 V evidences a partial oxidation of Mn as compared to 4.8 V.

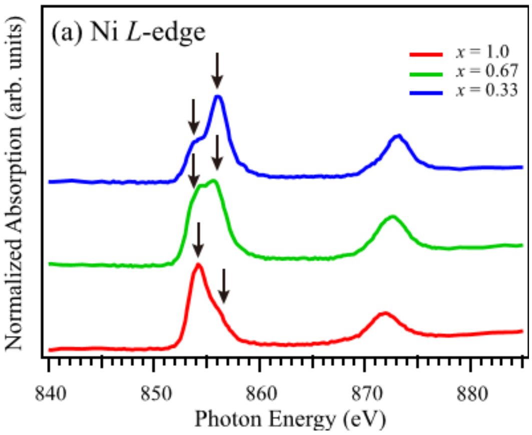

Figure S22. Experimental Ni L-edge XANES spectra of $Li_x(Ni_{1/3}Co_{1/3}Mn_{1/3})O_2$ measured in TEY mode (reproduced from Ref.[23S]).

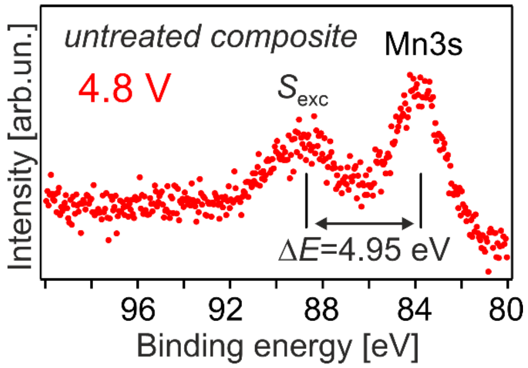

Figure S23. Mn 3s photoelectron spectra of the *untreated HE-NCM* cathode at charging state of 4.8 V versus Li^+/Li. ΔE is the exchange splitting defined as the energy difference between the main 3s photoemission and the exchange satellite, S_{exc}. ΔE =4.95 eV corresponds to oxidation state of Mn between +3.5 and 4+ (see Table II).

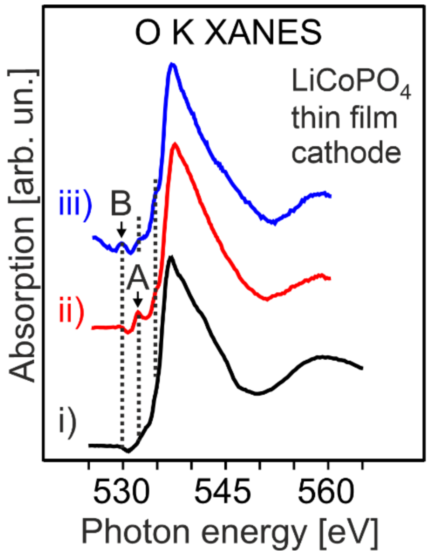

Figure S24. Evolution of O K XANES of $LiCoPO_4$ thin-film cathode: (i) as-prepared, (ii) charged to 5.1 V, (iii) after 1st electrochemical cycle with the upper cut-off of 5.1 V vs. Li^+/Li. The spectral features A=532.2 eV and B=529.9 eV are ascribed to Co 3d - O 2p hybridized states and O–C=O groups. The latter occur due to the electrolyte decomposition. The thin-film cathode does not contain a PVdF (binder) and conductive carbon.

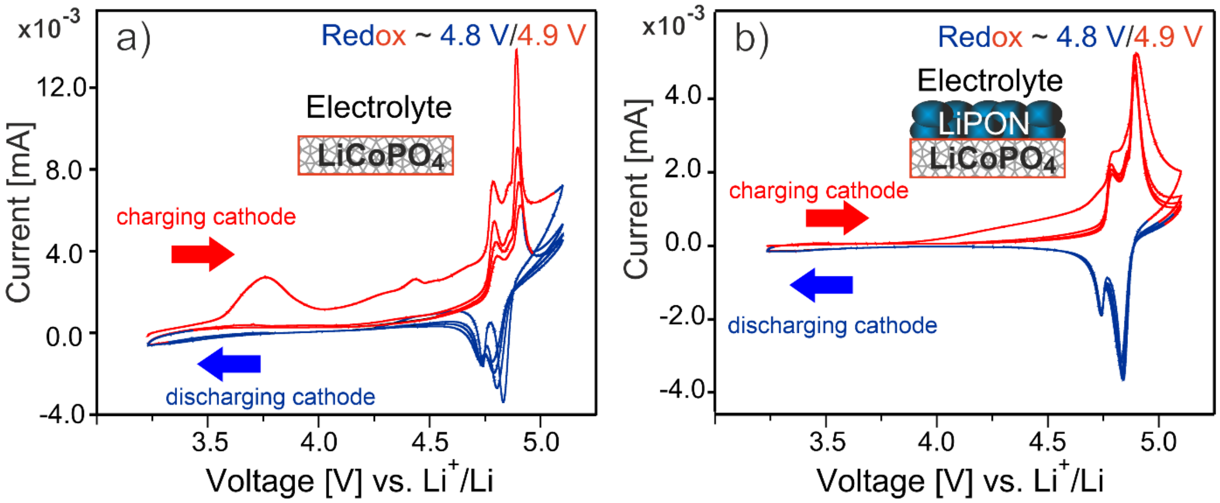

Figure S25. Cyclic voltammetry of $LiCoPO_4$ thin-film cathode: a) without coating, b) after coating with LIPON.

References

[1S] F. A. Susai, H. Sclar, S. Maiti, L. Burstein, O. Perkal, J. Grinblat, M. Talianker, S. Ruthstein, C. Erk, P. Hartmann, B. Markovsky, D. Aurbach, *ACS Appl. Energy Mater*. **2020**, *3*, 3609.

[2S] G. Cherkashinin, R. Eilhardt, S. Nappini, M. Cococcioni, I. Pís, S. dal Zilio, F. Bondino, N. Marzari, E. Magnano, L. Alff, *ACS Appl. Mater. Interfaces* **2022**, *14*, 543.

[3S] R. Qiao, Y.- D. Chuang, S. Yan, W. Yang, *Plos One* **2012**, *7*(11), e49182.

[4S] G. Cherkashinin, W. Jaegermann, *J. Chem. Phys*. **2016**, *144*, 184706.

[5S] E. M. Erickson, H. Sclar, F. Schipper, J. Liu, R. Tian, C. Ghanty, L. Burstein, N. Leifer, J. Grinblat, M. Talianker, J.- Y. Shin, J. K. Lampert, B. Markovsky, A. I. Frenkel, D. Aurbach, *Adv. Energy Mater.* **2017**, *7*, 1700708.

[6S] L. Martin, H. Martinez, D. Poinot, B. Pecquenard, F. Le Cras, *J. Phys. Chem. C.,* **2013**, *117*, 4421.

[7S] F. S. Gittleson, K. P. C. Yao, D. G. Kwabi, S. Y. Sayed, W. H. Ryu, Y. S.- Horn, A. D. Tayler, ChemElectroChem. **2015**, *2*, 1446.

[8S] K. Luo, M. R. Roberts, R. Hao, N. Guerrini, D. M. Pickup, Y.- S. Liu, K. Edström, J. Guo, A. V. Chadwick, L. C. Duda, P. G. Bruce, *Nature Chem*. **2016**, *8*, 684.

[9S] Q. Sun, Z. Wang, D. Wang, Z. Hong, M. Zhou, X. Li, *Catal. Sci. Technol*. **2018**, *8*, 4563.

[10S] H. P. Bonzel, G. Pirug, *Sur. Sci*. **1977**, *62*, 45.

[11S] A. Shiotari, H. Koshida, H. Okuyama, *Surf. Sci. Rep*. **2021**, *76*, 100500.

[12S] S. Maiti, H. Sclar, Rosy, J. Grinblat, M. Talianker, M. Tkachev, M. Tsubery, X. Wu, M. Noked, B. Markovsky, D. Aurbach, *Energy Storage Mater*. **2022**, *45*, 74.

[13S] K. Kubobuchi, M. Mogi, M. Matsumoto, T. Baba, C. Yogi, C. Sato, T. Yamamoto, T. Mizoguchi, H. Imai, *J. Appl. Phys*. **2016**, *120*, 142125.

[14S] W.- S. Yoon, M. Balasubramanian, K. Y. Chung, X.- Q. Yang, J. McBreen, C. P. Grey, D. A. Fischer, *J. Am. Chem. Soc*. **2005**, *127*, 17479.

[15S] J. García, J. Blasco, M. G. Proietti, M. Benfatto, *Phys. Rev. B.,* **1995**, *52*, 15823.

[16S] G. Cherkashinin, M. V. Lebedev, S. U. Sharath, A. Hajduk, S. Nappini, Elena Magnano, J. Mater. Chem. A., **2018**, *6*, 4966.

[17S] M. Mellin, Z. Liang, H. Sclar, S. Maiti, I. Pís, S. Nappini, E. Magnano, F. Bondino, I. Napal, R. Winkler, R. Hausbrand, J. P. Hofmann, L. Alff, B. Markovsky, D. Aurbach, W. Jaegermann, G. Cherkashinin, *Mater. Adv*. **2023**, *4*, 3746.

[18S] K. N. Wood, G. Teeter, *ACS Appl. Energy Mater*. **2018**, *1*, 4493.
[19S] E. Flores, P. Novák, E. J. Berg, *Front. Energy Research*. doi: 10.3389/fenrg.2018.0008.
[20S] V. S. Gorelik, D. Bi, Y. P. Voinov, A. I. Vodchits, B. P. Gorshunov, N. I. Yurasov, I. I. Yurasov, *IOP Conf. Ser.: J. Physics: Conf. Ser*. **2017**, *918*, 012035 doi:10.1088/1742-6596/918/1/012035.
[21S] H. Sclar, S. Maiti, R. Sharma, E. M. Erickson, J. Grinblat, R. Raman, M. Talianker, M. Noked, A. Kondrakov, B. Markovsky, D. Aurbach, *Inorganics* **2022**, *10*, 1. https://doi.org/10.3390/inorganics10030039.
[22S] J. Zheng, W. Shi, M. Gu, J. Xiao, P. Zuo, C. Wang, J.- G. Zhang, *J. Electrochem. Soc.* **2013**, *160*, A2212.
[23S] K. Kubobuchi, M. Mogi, M. Matsumoto, T. Baba, C. Yogi, C. Sato, T. Yamamoto, T. Mizoguchi, H. Imai, *J. Appl. Phys*. **2016**, *120*, 142125.
